# Promoting exsolution of RuFe alloy nanoparticles on Sr_2_Fe_1.4_Ru_0.1_Mo_0.5_O_6−*δ*_ via repeated redox manipulations for CO_2_ electrolysis

**DOI:** 10.1038/s41467-021-26001-8

**Published:** 2021-09-27

**Authors:** Houfu Lv, Le Lin, Xiaomin Zhang, Rongtan Li, Yuefeng Song, Hiroaki Matsumoto, Na Ta, Chaobin Zeng, Qiang Fu, Guoxiong Wang, Xinhe Bao

**Affiliations:** 1 State Key Laboratory of Catalysis, Dalian National Laboratory for Clean Energy, Dalian Institute of Chemical Physics, Chinese Academy of Sciences, Dalian, P. R. China; 2 University of Chinese Academy of Sciences, Beijing, P. R. China; 3 School of Physical Science and Technology, ShanghaiTech University, Shanghai, P. R. China; 4 Hitachi High-tech (Shanghai) Co., Ltd, Shanghai, P. R. China

**Keywords:** Electrocatalysis, Heterogeneous catalysis, Fuel cells

## Abstract

Metal nanoparticles anchored on perovskite through in situ exsolution under reducing atmosphere provide catalytically active metal/oxide interfaces for CO_2_ electrolysis in solid oxide electrolysis cell. However, there are critical challenges to obtain abundant metal/oxide interfaces due to the sluggish diffusion process of dopant cations inside the bulk perovskite. Herein, we propose a strategy to promote exsolution of RuFe alloy nanoparticles on Sr_2_Fe_1.4_Ru_0.1_Mo_0.5_O_6−*δ*_ perovskite by enriching the active Ru underneath the perovskite surface via repeated redox manipulations. In situ scanning transmission electron microscopy demonstrates the dynamic structure evolution of Sr_2_Fe_1.4_Ru_0.1_Mo_0.5_O_6−*δ*_ perovskite under reducing and oxidizing atmosphere, as well as the facilitated CO_2_ adsorption at RuFe@Sr_2_Fe_1.4_Ru_0.1_Mo_0.5_O_6−*δ*_ interfaces. Solid oxide electrolysis cell with RuFe@Sr_2_Fe_1.4_Ru_0.1_Mo_0.5_O_6−*δ*_ interfaces shows over 74.6% enhancement in current density of CO_2_ electrolysis compared to that with Sr_2_Fe_1.4_Ru_0.1_Mo_0.5_O_6−*δ*_ counterpart as well as impressive stability for 1000 h at 1.2 V and 800 °C.

## Abstract (web summary)

Metal nanoparticles anchored on perovskite provide catalytically active interfaces for CO2 electrolysis. The authors promote exsolution of RuFe alloy nanoparticles on Sr2Fe1.4Ru0.1Mo0.5 O6−δ perovskite by enriching the active Ru underneath the perovskite surface via repeated redox manipulations.

## Introduction

Solid oxide electrolysis cell (SOEC) for CO_2_ electrolysis can efficiently convert renewable electricity into chemical energy as stored in CO^1–4^. Perovskite and its derivatives have been investigated as one of the most promising SOEC cathode materials due to their considerable catalytic activity and stability^5^. Redox exsolution on perovskite is a feasible strategy to manipulate catalytic active sites, in which catalytically active transition metals such as Rh, Pd, Pt, Fe, Ni, Co, etc. are substituted in the perovskite lattice as solid solution under an oxidizing atmosphere, and then exsolved as metal nanoparticles (NPs) anchored on the perovskite surface under reducing atmosphere^6–10^. The perovskites undergoing redox exsolution exhibit unique catalytic activity, thermal stability, and coking resistance in SOEC, solid oxide fuel cell (SOFC), and other heterogeneous catalytic reactions^11–16^.

The perovskite used in SOEC and SOFC has a large crystalline size of several hundred nanometers due to high-temperature (>1000 °C) calcination during powder preparation and cell fabrication^17,18^. The exsolution process occur initially within the bulk perovskite with uniformly doped active metal cations^19,20^, and only a limited proportion of metal NPs can be exsolved onto the perovskite surface under reducing atmosphere due to the sluggish diffusion process of dopant B-site cations inside the perovskite lattice^17,21–23^. Several strategies have been developed to promote the exsolution of metal NPs such as A-site defect^18,24,25^, phase transformation engineering^26,27^, and topotactic ion exchange^28,29^, etc. These strategies indeed facilitate the exsolution process, however, the critical issue of long diffusion distance remains unresolved. Ideal exsolution may necessitate the enrichment of the dopant B-site cations underneath the perovskite surface, which would facilitate the exsolution on the surface without the need of diffusion from the bulk^21,30^. Therefore, developing a reliable strategy for surface engineering regulation would provide more viable options for promoting the in situ growth of highly active and stable metal/oxide interfaces.

Herein, we propose a strategy to promote the exsolution of RuFe alloy NPs on Sr_2_Fe_1.4_Ru_0.1_Mo_0.5_O_6−*δ*_ (SFRuM) perovskite by enriching the active Ru underneath the surface via repeated redox manipulations. The atomic-scale dynamic structure evolution of SFRuM perovskite under the reducing and oxidizing atmosphere is investigated using in situ scanning transmission electron microscopy (STEM) with energy-dispersive X-ray spectroscopy (EDS) and electron energy loss spectrum (EELS), in combination with in situ X-ray diffraction (XRD) and density functional theory (DFT) calculations. Under reducing atmosphere, Ru atoms in SFRuM are preferentially exsolved to form Ru NPs anchored on the SFRuM surface, and then Fe atoms migrate onto the surface of Ru NPs to generate RuFe intermetallic alloy. Under oxidizing atmosphere, Fe atoms in RuFe alloy are preferentially oxidized and move back to the perovskite, and subsequently, Ru atoms migrate back to surface-biased B-sites. Fe species can reversibly serve as a “templating agent” drawing Ru atoms out of the perovskite. The repeated redox manipulations result in the enrichment of Ru underneath the SFRuM perovskite surface and thus facilitate abundant exsolution of RuFe alloy NPs under reducing atmosphere. The density of exsolved RuFe alloy NPs reaches ~21,000 particles μm^−2^ after four redox manipulations, which is ~3.6 times as much as that after the first reduction treatment while the size of RuFe alloy NPs maintains similar. The in situ grown RuFe@SFRuM interfaces facilitate CO_2_ adsorption and boost CO_2_ electrolysis performance in SOEC with impressive stability for 1000 h.

## Results

### In situ STEM and crystalline structure characterization

The crystalline structures of the as-prepared, reduced, reoxidized, and re-reduced SFRuM were examined using XRD and transmission electron microscopy (TEM) (Supplementary Fig. 1). As-prepared SFRuM shows a double perovskite structure. The reduced sample presents a mixture of the Ruddlesden-Popper layered perovskite (RP-SFRuM) with RuFe alloy phase (SFRuM R1 and RuFe@SFRuM), indicating that the phase transition and exsolution of RuFe alloy occurs during the reduction process. The crystalline structure reconstructs to the initial state after reoxidation (SFRuM O1), and RuFe alloy could be exsolved again after re-reduction (SFRuM R2), which shows a characteristic self-regeneration function^11,12,31^.

**Fig. 1:**
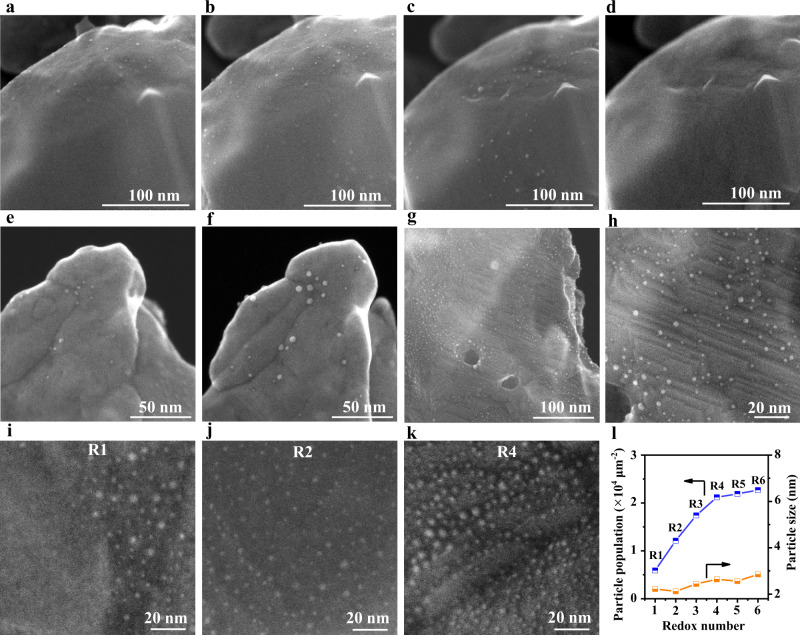
Morphological study of the SFRuM catalysts. In situ secondary electron (SE)-STEM images of SFRuM after reduction at 800 °C for ~15 min (**a**), after reduction at 800 °C for ~30 min (**b**), after reoxidation at 800 °C for ~30 min (**c**), and after reoxidation at 800 °C for ~40 min (**d**). In situ SE-STEM images of SFRuM O1 after reduction at 600 °C for ~10 min (**e**), after reduction at 800 °C for ~10 min (**f**), and after reduction at 800 °C for ~60 min (**g**). **h** Magnified image of (**g**). Ex situ SE-STEM images of SFRuM R1 (**i**), SFRuM R2 (**j**), and SFRuM R4 (**k**). **l** Population and size distribution of metal NPs exsolved after different redox manipulations.

Using in situ aberration-corrected STEM with a secondary electron detector, we are able to conduct surface morphology evolution during redox manipulations. The perovskite initially exhibited a pristine surface with no existence of exsolved metal NPs after reduction at 600 °C (Supplementary Fig. 2b). A few small-size metal NPs emerged until the reduction temperature reached 800 °C (Fig. 1a), which were apparently present on the surface after continuous reduction for ~30 min (Fig. 1b). Under oxidizing atmosphere, metal NPs were oxidized with a large size and then redissolved into the perovskite completely (Fig. 1c, d and Supplementary Fig. 2). In situ STEM results confirm that Ru in SFRuM repeats the cycle of forming a solid solution and segregation as metal NPs during redox manipulations. Subsequently, SFRuM O1 was also investigated under reducing atmosphere by in situ STEM and different phenomena were observed, in which metal NPs could be exsolved after reduction at 600 °C (Fig. 1e and Supplementary Fig. 3). Abundant metal NPs were present on the surface after increasing the reduction temperature to 800 °C (Fig. 1f–h), and the NPs can be observed mainly on the steps of the perovskite, where the presence of crystallographic defects can act as nucleation sites^18,20^ (Supplementary Fig. 3i–k). Metal NPs were easier to be exsolved on SFRuM O1, corroborating different segregation properties associated with the variation in surface composition.

To provide insight into the difference in exsolution, the correlation between the redox manipulations and the number of exsolved metal NPs was investigated (Fig. 1i–l and Supplementary Fig. 4). In the case of the SFRuM R1, a limited fraction of B-site cations could be exsolved (Fig. 1i). In contrast, much more exsolved metal NPs were observed on redox manipulated samples (Fig. 1j, k and Supplementary Fig. 4). The relationship between the density of the exsolved metal NPs and the number of the redox cycles is shown in Fig. 1l, and the density of exsolved metal NPs increases with the number of redox cycles. The density of exsolved metal NPs reaches ~21,000 particles μm^−2^ after four redox manipulations, which is ~3.6 times as much as that of SFRuM R1. Notably, the redox manipulations do not affect the Fe/Ru ratio and the size of the exsolved metal NPs (Fig. 1l and Supplementary Fig. 5). In consequence, exsolution is greatly promoted through repeated redox manipulations, which is probably due to Ru enrichment underneath the SFRuM surface.

**Fig. 2:**
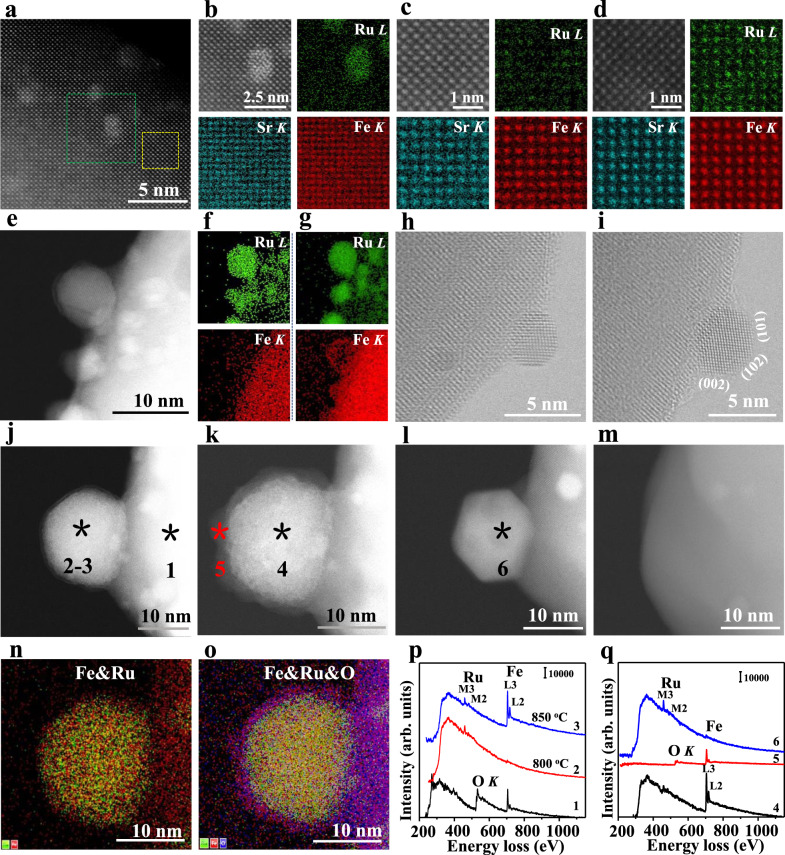
In situ STEM, STEM-EDS, and STEM-EELS results. **a** HAADF-STEM image of SFRuM R1 and the atomic-scale elemental maps, circled with a green dotted line **b** and a yellow dotted line **c** (010). **d** HAADF-STEM image of SFRuM O1 and the atomic-scale elemental maps (010). **e** In situ DF-STEM image and STEM-EDS elemental maps of SFRuM at 800 °C for ~15 min (**f**) and then at 850 °C for ~15 min (g). In situ BF-STEM images after reduction at 800 °C for ~15 min (**h**) and then at 850 °C for ~15 min (**i**). In situ DF-STEM images of SFRuM after reduction at 800 °C for ~60 min and 850 °C for ~30 min (**j**), after reoxidation at 200 °C for ~70 s (**k**), after reoxidation at 800 °C for ~30 min (**l**), and after reoxidation at 800 °C for ~60 min (**m**). **n**, **o** The STEM-EDS elemental maps of **j** and **k**. **p**, **q** In situ STEM-EELS spectra in **j**–**l**.

To investigate the crystalline structure and composition of the perovskite after reduction (SFRuM R1) and after reoxidation (SFRuM O1), we examined the samples using atomic-scale STEM and STEM-EDS elemental maps. As shown in the high angle annular dark field (HAADF)-STEM image of SFRuM R1 (Fig. 2a), metal NPs were exsolved from the parent perovskite. Atomic-scale STEM-EDS elemental maps were conducted in the parent perovskite with and without metal NPs to investigate the ordering and positions of Ru (Fig. 2b, c and Supplementary Fig. 6). Ru signals were not clearly observed in the perovskite except in the metal NPs (Fig. 2c), suggesting that part of the Ru was exsolved into the metal NPs (Fig. 2b). Meanwhile, Ru signals were clearly observed in the positions of Fe and Mo for SFRuM O1, which implies the complete dissolution of Ru into the B-sites of perovskite after reoxidation (Fig. 2d and Supplementary Fig. 7).

The dynamic evolution upon exsolution and dissolution of RuFe alloy NPs was investigated by in situ STEM to reveal the mechanism underlying the surface enrichment of Ru. In situ dark field (DF)-STEM images and STEM-EDS elemental maps were collected during the reduction process, and Ru-rich metal NPs were exsolved at the preliminary stage at 800 °C (Fig. 2e, f and Supplementary Fig. 8a). During the assembling process of RuFe alloy NPs, reduced Fe was observed to migrate onto the surface of Ru NPs through the Ru@SFRuM interface (Fig. 2g and Supplementary Figs. 8,14), which is similar to the formation process of strong metal-support interaction^32^. The transient overlayer structure is the intermediate state, and RuFe alloy NPs could be assembled after reduction at 800 °C for ~60 min and then at 850 °C for ~30 min (Fig. 2j, n and Supplementary Fig. 9), which was also validated by ex situ EDS results (Supplementary Figs. 10,11). In addition, epitaxially oriented metal NPs evolution remains isotropic, growing proportionally in both height and width simultaneously^33^ (Fig. 2h, i and Supplementary Figs. 12, 13), especially during the migration of Fe onto the Ru NPs (Supplementary Fig. 14).

The evolution of RuFe alloy NPs under an oxidation atmosphere was also investigated (Supplementary Fig. 15). As illustrated by the in situ DF-STEM, STEM-EDS, and STEM-EELS results in Fig. 2j, n, p, metallic Ru and Fe were uniformly distributed in the exsolved metal NPs, demonstrating the formation of a RuFe alloy phase. A structure of FeO_x_ shell and RuFe alloy core was well reflected after in situ reoxidation in 10 Pa of O_2_ at 200 °C for ~70 s (Fig. 2k, o, q, Supplementary Fig. 9 and Supplementary Movie 1), in line with ex situ STEM-EDS results (Supplementary Figs. 10, 16, 17). After exposure to the O_2_ atmosphere at 800 °C for 30 min, the metal NPs evolved into a regular shape (Fig. 2l and Supplementary Figs. 18, 19). Changes in the constituent of RuFe metal NPs into Ru-rich metal NPs could be ascribed to the prior oxidation and dissolution of FeO_x_ into the perovskite (Fig. 2q). As a manifestation of the self-regeneration function, the Ru-rich metal NPs dissolved into the perovskite completely after exposure to the O_2_ atmosphere for 60 min at 800 °C (Fig. 2m and Supplementary Figs. 15, 19). In situ STEM, STEM-EDS, and STEM-EELS results demonstrate the priority exsolution but the hysteresis dissolution of Ru in RuFe alloy NPs, which reveals the formation mechanism of Ru enrichment underneath the SFRuM surface.

### Crystal structure evolution and DFT calculations

**Fig. 3:**
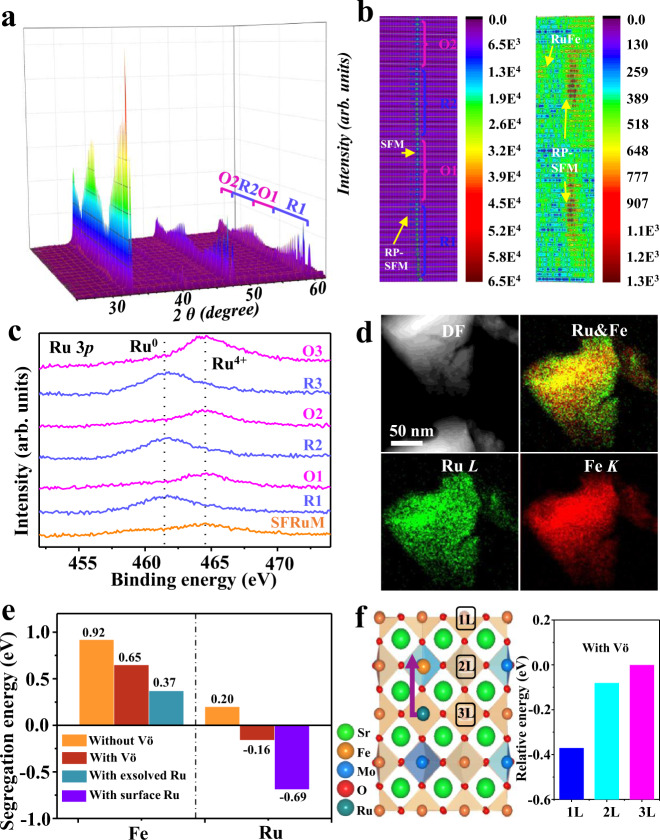
Characterization and DFT results of SFRuM. **a** In situ XRD patterns of as-prepared SFRuM upon switching between reducing and oxidizing atmosphere at 800 °C. **b** Depth profile of XRD patterns at 31–33° and 43–45° of (**a**). **c** Ru 3*p* XPS spectra of SFRuM after redox manipulations. **d** STEM-EDS elemental maps of SFRuM O3. **e** Comparison of segregation and co-segregation energies with Fe and the doped Ru. **f** Schematic of exsolution process with relative energy of the slab as a function of Ru positions. 1L denotes the surface layer and 3L corresponds the inner layer.

In situ XRD experiments were carried out to monitor the structural evolution during redox manipulations at 800 °C (Fig. 3a, b). Layered perovskite with RuFe alloy phases emerged during reduction and vanished during reoxidation. With increasing reduction time in the first reduction step, a weak phase transformation and RuFe alloy phase emerged, and the mixed structure evolved back to the original perovskite form after reoxidation. Similar observations were acquired during the second redox cycle, except that the characteristic peak intensity of SFRuM was weakened, whereas the characteristic peak intensity of emergent RP phase perovskite and RuFe alloy was enhanced (Fig. 3a, b). This phenomenon is consistent with the behaviors observed in situ (Fig. 1e–h) and ex situ (Fig. 1i–k and Supplementary Fig. 4) SE-STEM results that promoted exsolution was triggered for the reoxidized SFRuM. SFRuM shows unexpected but reasonable redox self-regeneration functions, which is due to surface enrichment of Ru as mentioned above.

The changes in chemical states and components of surface-biased elements were demonstrated by X-ray photoemission spectroscopy (XPS) analysis. Note that the C 1*s* and Ru 3*d* photoemissions overlap, hence Ru 3*p* spectra were collected. Peak intensity of Ru^4+^ in the as-prepared SFRuM with the binding energy of 464.2 eV is relatively weak^34^, while metallic Ru^0^ species with the binding energies of 461.3 eV can be observed for SFRuM R1 with high intensity. After reoxidation (SFRuM O1), the same chemical environment with SFRuM demonstrates redox recyclability and the enhanced intensity of Ru 3*p*_3/2_ spectrum displays an increase of Ru amount on the perovskite surface of SFRuM O1^34^. According to the semiquantitative XPS analysis, a significant increase in the atomic Ru/Sr ratio from 0.053 to 0.12 can be observed after three redox manipulations (Fig. 3c and Supplementary Table 1), indicative of gradual enrichment of Ru amount on the surface via redox manipulations. To gain direct insights into the componential change, we performed STEM-EDS analysis. The representative elemental maps clearly show a homogeneous distribution of Ru in SFRuM, whereas SFRuM O3 substantiates the formation of a core-shell structure, demonstrating the formation of Ru-enriched surface through microcosmic elemental segregation (Fig. 3d and Supplementary Figs. 20, 21), which confirms the self-enrichment function of SFRuM after repeated redox manipulations.

The sequential exsolution of Ru and Fe and surface enrichment of Ru are further elaborated by DFT calculations^14^ (Fig. 3e, f and Supplementary Fig. 22). The calculated co-segregation energies of Ru and Fe with oxygen vacancy (${{{{{{\rm{V}}}}}}}_{{{{{{\rm{O}}}}}}}^{{{{{{\rm{\cdot \cdot }}}}}}}$) are −0.16 and 0.65 eV, respectively, which are lower than those without ${{{{{{\rm{V}}}}}}}_{{{{{{\rm{O}}}}}}}^{{{{{{\rm{\cdot \cdot }}}}}}}$, indicative of the critical role of ${{{{{{\rm{V}}}}}}}_{{{{{{\rm{O}}}}}}}^{{{{{{\rm{\cdot \cdot }}}}}}}$ in the exsolution^22,25,30^. The lower segregation energy illustrates that Ru has a higher tendency to be exsolved than Fe. Ru exsolution to the surface could decrease the segregation energy of Fe to 0.37 eV and thus facilitates Fe exsolution to form RuFe alloy^35^, which is in good agreement with the in situ STEM-EDS results (Fig. 2f, g). Figure 3f displays the calculated total energies of SFRuM slabs with surface-segregated and solid-solution states of Ru atoms. The surface-segregated state is slightly more stable than the solid-solution one. Therefore, the exsolution may originate from the preferential migration of Ru to form Ru metal NPs^28,29^. Subsequently, Fe atoms migrate to the Ru metal NPs to form RuFe alloy NPs (Fig. 2g, j). Upon subsequent exposure to the O_2_ atmosphere, Fe moves back to the bulk perovskite preferentially, and subsequently Ru migrates back to the surface-biased B-sites. Ru is pulled out from perovskite bulk gradually through such a cyclic process and consequently forms a Ru-enriched surface on the perovskite. After surface enrichment of Ru, the co-segregation energy of Ru with ${{{{{{\rm{V}}}}}}}_{{{{{{\rm{O}}}}}}}^{{{{{{\rm{\cdot \cdot }}}}}}}$ could be further reduced to −0.69 eV, and thus promotes the exsolution of more RuFe alloy NPs (Fig. 3e), consistent with in situ (Fig. 1e–h) and ex situ (Fig. 1i–k and Supplementary Fig. 4) SE-STEM results. Redox manipulations would result in enrichment of dopant Ru cations underneath the perovskite surface, which is conducive to promote the exsolution of RuFe alloy NPs.

### Electrochemical performance

**Fig. 4:**
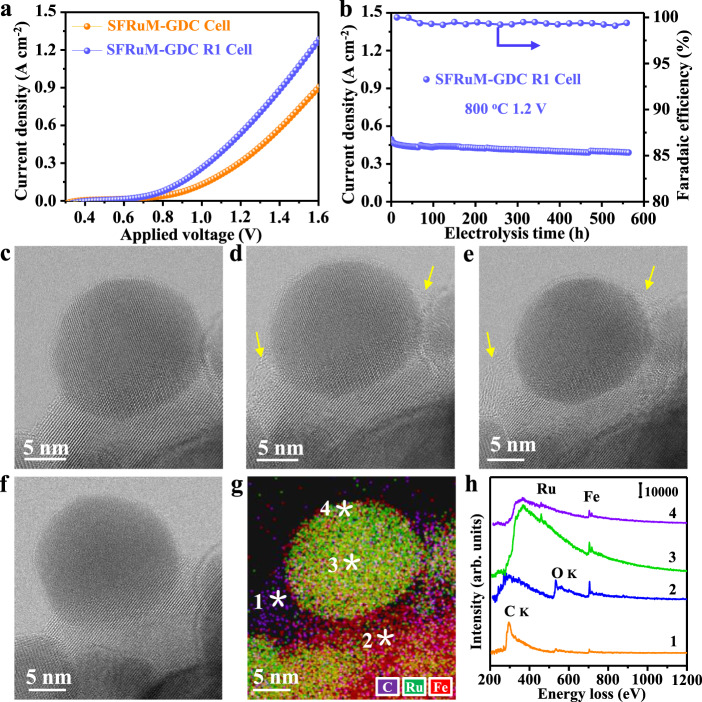
CO_2_ electrolysis and in situ STEM results. **a** I–V curves of SFRuM-GDC and SFRuM-GDC R1 cells at 800 °C. **b** Stability test of SFRuM-GDC R1 cell and CO Faradaic efficiencies at 800 °C and 1.2 V. In situ BF-STEM images of SFRuM after in situ reduction at 800 °C for ~1 h (**c**), after exposed to 10 Pa of CO_2_ for 10 min at 200 °C (**d**), after exposed to 10 Pa of CO_2_ for 30 min at 200 °C (**e**) (The adsorbed species are indicated by yellow arrows), and after heating up to 800 °C under vacuum (**f**). **g**, **h** In situ STEM-EDS map and in situ STEM-EELS spectra of (**e**).

Figure 4a and Supplementary Fig. 23 show CO_2_ electrolysis performance using La_0.8_Sr_0.2_Ga_0.8_Mg_0.2_O_3−*δ*_ (LSGM) electrolyte supported electrolysis cell. SFRuM perovskite phase and Gd_0.2_Ce_0.8_O_1.9_ (GDC) fluorite phase were used as the composite cathode (SFRuM-GDC). As-prepared SFRuM-GDC cell exhibited relatively low current density as Ru is less exposed onto the surface. High CO_2_ electrolysis performance can be achieved for the SFRuM-GDC R1 cell, with a few active RuFe alloy NPs exsolved on the surface (Fig. 1i). The stability of the SFRuM-GDC R1 cell for CO_2_ electrolysis was examined at 800 °C and 1.2 V (Fig. 4b). There was a slight drop in the primary stage and the current density showed no observable degradation for 567 h. No coking was confirmed by the calculated CO Faradaic efficiencies (Fig. 4b) and Raman results (Supplementary Fig. 24). Conventionally, these anchored metal NPs with preferential orientations towards the perovskite support will maintain the active interfaces under reaction conditions (Supplementary Figs. 13, 16)^36–39^. The current density of 2.25 A cm^−2^ was obtained using a thin LSGM electrolyte that exceeds most state-of-the-art electrolyzers (Supplementary Fig. 25 and Table 2), benefiting from the highly active metal-perovskite interfaces^40,41^.

To directly observe and obtain more detailed information, we conducted in situ STEM on the dynamic evolution of the RuFe@SFRuM interface under the CO_2_ atmosphere. A stable and clean RuFe@SFRuM interface could be obtained after in situ reduction at 800 °C (Fig. 4c and Supplementary Fig. 26). Upon exposure to CO_2_ atmosphere at 200 °C, a gradual decoration of the interface with an amorphous material of low-contrast was observed (yellow arrow in Fig. 4d, e, Supplementary Figs. 27, 28, and Supplementary Movie 2). STEM-EDS elemental maps and STEM-EELS spectra (Fig. 4g, h) reveal that these species contain carbon and oxygen, which are more abundant than that on the surface of RuFe alloy NPs and the parent perovskite. These results indicate that RuFe@SFRuM interfaces facilitate CO_2_ adsorption compared to either RuFe alloy NPs or parent perovskite alone. After heating up to 800 °C under vacuum, adsorbed species disappear probably through CO or CO_2_ desorption (Fig. 4f). The in situ approach enables to derive a holistic view under the reaction conditions that is important for the understanding of the RuFe@SFRuM interface characteristics.

**Fig. 5:**
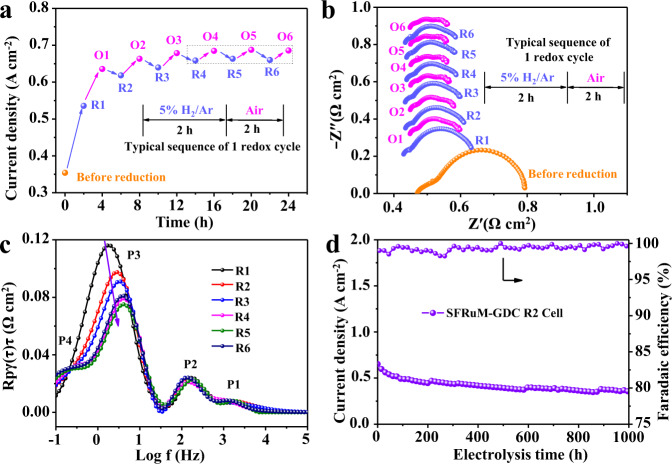
Electrochemical results. **a** CO_2_ electrolysis performance of SFRuM-GDC cell after six redox cycles at 800 °C and 1.2 V. **b** The corresponding in situ EIS results. **c** DRT spectra of the RuFe alloy NPs decorated cells calculated from **b**. **d** Stability test of SFRuM-GDC R2 cell and CO Faradaic efficiencies at 800 °C and 1.2 V.

Ru-containing SFRuM perovskite catalyst can function as a self-regeneration and surface Ru enrichment catalyst via redox manipulations. The electrochemical performance of the preceding SFRuM after every redox manipulation was subsequently assessed for CO_2_ electrolysis. The measurement was carried out sequentially over six repetitions of redox cycles. CO_2_ electrolysis performance was improved after the first reduction treatment, known as the decoration of RuFe alloy NPs. Performance improvement can be further obtained for SFRuM-GDC O1 cell, mainly due to the atomically dispersed lattice confined Ru sites^34,42^. The population of the exsolved RuFe alloy NPs increases with the cycle of redox manipulations due to the surface enrichment of Ru (Fig. 1l), which resulted in a gradual enhancement of CO_2_ electrolysis performance. Compared to SFRuM-GDC cell, the enhancement in current density for CO_2_ electrolysis is 51.3% for SFRuM-GDC R1 cell, 74.6% for SFRuM-GDC R2 cell, and finally reaches 86.4% for SFRuM-GDC R6 cell at 800 °C and 1.2 V (Fig. 5a). The current density was improved during every reduction process until after four redox manipulations, and then could be reproducible on/off on a regular basis^43,44^ (Fig. 5a), clearly revealing the structure-activity relationship. The surface characteristics were greatly changed with the surface enrichment of Ru via redox manipulations, promoting the exsolution of abundant RuFe alloy NPs and facilitating the CO_2_ electrolysis.

Electrochemical impedance spectroscopy (EIS) can provide operando insights into the switching of active sites for CO_2_ electrolysis in both reducing and oxidizing atmospheres (Fig. 5b). The high-frequency intercept of EIS with the real axis represents the ohmic resistance (*R*_o_) mainly from the electrolyte, and the distance between the highest and lowest frequency intercepts of EIS with the real axis are related to the electrode polarization resistance (*R*_p_). Periodic diminution of *R*_o_ demonstrates the enhanced conductivity, which can be attributed to the synergetic effect of the exsolved metal NPs, the RP phase perovskite through a phase transition, and the increased oxygen vacancies during the exsolution^45^. And the variation tendency of the total resistance corresponds to the CO_2_ electrolysis performance in Fig. 5a. The EIS can separate into several elementary electrode processes through distribution function of relaxation times (DRT, Supplementary Figs. 29, 30) analysis, including ion migration process (P1), oxygen evolution reaction at the anode (P2), electrochemical adsorption and activation process (P3), and gas diffusion process (P4) from high to low frequency range^46–48^. Generally, the resistance of the P3 process dominates CO_2_ electrolysis kinetics^31,49^, which strongly depends on the catalytic active sites. The decreasing peak area of the P3 process after reduction and re-reduction processes takes responsibility for the gradually enhanced CO_2_ electrolysis performance (Fig. 5a, c), which is an essential manifestation of gradually promoted exsolution of RuFe alloy NPs. Compared with those of other perovskite electrodes, SFRuM shows the lowest polarization resistance of 0.11 Ω cm^−2^ at 800 °C and 1.2 V under similar test conditions (Supplementary Table 3), revealing a great improvement in the catalytic activity through redox manipulations. In addition, the stability of the exsolved NPs obtained via redox manipulations was evaluated for SFRuM-GDC R2 cell (Fig. 5d), and the CO_2_ electrolysis performance remained relatively stable for 1000 h, validating the impressive activity, stability, and renewability of the metal/oxide interface (Supplementary Fig. 31). In situ redox manipulations to regenerate catalysts and promote the exsolution of abundant metal NPs are crucial for the perovskite-based catalysts, which could serve as a general strategy for other exsolution systems.

### Theoretical simulation

**Fig. 6:**
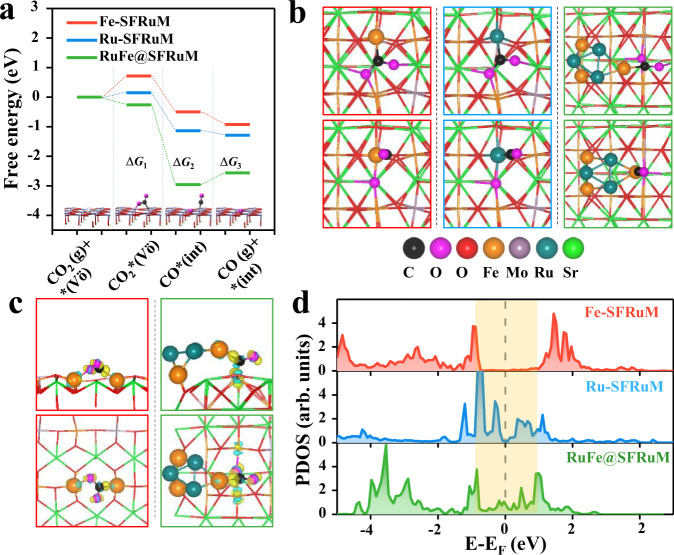
DFT-based energetic and electronic structure analysis of CO_2_ electrolysis. **a** Gibbs free energy diagram for CO_2_ electrolysis over three active sites: Fe and Ru centers at SFRuM (121) surface and Fe border at RuFe@SFRuM interface (800 °C and −1.0 V). *(${{{{{{\rm{V}}}}}}}_{{{{{{\rm{O}}}}}}}^{{{{{{\rm{\cdot \cdot }}}}}}}$) and *(int) denote the oxygen-defected and the intact surfaces. **b** Corresponding local atomic configurations (top view) from CO_2_* (upper) to CO* (lower). **c** Top (upper) and side (lower) views of electron density differences of CO_2_ adsorption over Fe-SFRuM surface and RuFe@SFRuM interface. Yellow and cyan areas denote the accumulation and dissipation of electrons. **d** PDOS of active Fe and Ru centers at SFRuM surface and RuFe@SFRuM interface before CO_2_ adsorption. The dashed gray line marks the Fermi level and the orange block highlights the main difference of the *d*-band.

DFT calculations provide insights into the essence of reactivity differences (see “Methods” and Supplementary Figs. 32−34 for details). CO_2_ activation and oxygen migration are the key issues to be taken into account. We focus on the CO_2_ activation process as SFM and RP-SFM perovskites possess similar O^2−^ migration ability^31^. The CO_2_ activation process involves three elementary steps: CO_2_ adsorption (Δ*G*_1_), dissociation (Δ*G*_2_), and CO desorption (Δ*G*_3_). As shown in Fig. 6a, Δ*G*_1_ is 0.72 eV on the Fe site of SFRuM surface (Fe-SFRuM), which is −0.26 eV at the Fe-terminated interface (RuFe@SFRuM) through a bidentate-CO_2_* formation with the C-Fe bonding and O (of CO_2_) trapping at the ${{{{{{\rm{V}}}}}}}_{{{{{{\rm{O}}}}}}}^{{{{{{\rm{\cdot \cdot }}}}}}}$ (Fig. 6b)^50^. These CO_2_* species subsequently dissociate to CO* through an electrochemical process with 2e^−^ transfer^47^. For the third elementary step, CO* desorption energies (Δ*G*_3_) are −0.43 and 0.39 eV for Fe-SFRuM and RuFe@SFRuM, respectively. The rate-determining step is CO_2_ adsorption (Δ*G*_1_ = 0.72 eV) for Fe-SFRuM and CO desorption (Δ*G*_3_ = 0.39 eV) for RuFe@SFRuM, therefore, the latter is relatively favorable and represents a high activity. Figure 6c shows that electron transfer mainly occurs between C and Fe, indicative of a pivotal role of Fe in CO_2_ electrolysis. In the light of projected density of states (PDOS) (Fig. 6d), Fe at RuFe@SFRuM interface possesses richer densities (the orange block highlights) around the Fermi level and a higher *d*-band center (*ε*_*d*_ = −1.58) compared to SFRuM surface (*ε*_*d*_ = −1.76), indicative of strong bonding of the metal/oxide interface to CO_2_. We also note that the interface can provide a highly O-constrained ${{{{{{\rm{V}}}}}}}_{{{{{{\rm{O}}}}}}}^{{{{{{\rm{\cdot \cdot }}}}}}}$ (estimated by the ${{{{{{\rm{V}}}}}}}_{{{{{{\rm{O}}}}}}}^{{{{{{\rm{\cdot \cdot }}}}}}}$ formation energy, *E*_ovf_ = −1.14 eV) as compared to the surface (*E*_ovf_ = 0.36 eV). Therefore, we suggest that the RuFe@SFRuM interface boosts the CO_2_ electrolysis, benefiting from the synergetic effect of the interface confined Fe and the unique oxygen vacancy. Additionally, Fe-terminated (RuFe@SFRuM) and Ru-terminated (FeRu@SFRuM) interface models exhibit a significantly different adsorbability for CO, implying interface termination engineering is also a strategy targeted for tuning the performance (Supplementary Fig. 35).

With regard to the surface-enrichment effect of Ru, the comparison is made between the isolated Ru site on the SFRuM surface (Ru-SFRuM) and the pristine Fe-SFRuM. CO_2_ electrolysis should mainly lie at the electronic effect owing to the difference between 3*d*-block Fe and 4*d*-block Ru^51^. In Fig. 6a, CO_2_ adsorption on Ru-SFRuM is enhanced by 0.57 eV relative to that on Fe-SFRuM, indicating that CO_2_ is well activated by the isolated Ru site. Further PDOS analysis in Fig. 6d shows that the isolated Ru exhibits more enriched *d*-band states around the Fermi level than Fe, causing an intensified interaction between Ru and CO_2_. Such distinctive electronic structure of Ru-SFRuM improves the activity of CO_2_ electrolysis versus that of Fe-SFRuM, which is consistent with the improved CO_2_ electrolysis performance by surface enriched Ru in Fig. 5a. Periodic oscillation in CO_2_ electrolysis performance depends upon dynamic switching between the exsolved RuFe alloy NPs and dispersive Ru sites on SFRuM.

## Discussion

In situ microscopy and spectroscopy characterizations reveal dynamic exsolution and dissolution processes of RuFe alloy NPs on SFRuM surface at the atomic scale, as well as Ru enrichment on SFRuM surface upon multiple exsolution and dissolution of Ru and Fe after repeated redox manipulations, which significantly promotes the exsolution of abundant RuFe alloy NPs on SFRuM surface. The density of exsolved RuFe alloy NPs reaches ~21,000 particles μm^−2^ after four redox manipulations, which is ~3.6 times as much as that after the first reduction treatment while the size of RuFe alloy NPs maintains similar. The in situ grown RuFe@SFRuM interfaces show highly active and stable electrochemical performance for CO_2_ electrolysis in SOEC, as supported by EIS analysis and DFT calculations. Our study provides a strategy to obtain abundant catalytically active metal/oxide interfaces on perovskites for CO_2_ electrolysis in SOEC and other heterogeneous catalytic reactions via repeated redox manipulations.

## Methods

### Chemicals

Sr(NO_3_)_2_ (AR, Aladdin Industrial Corporation), Fe(NO_3_)_3_·9H_2_O (AR, Aladdin Industrial Corporation), (NH_4_)_6_Mo_7_O_24_·4H_2_O (AR, Sinopharm Chemical Reagent), RuCl_3_ (AR, Tianjin Jinbolan Fine Chemical), glycine (AR, Sinopharm Chemical Reagent), citric acid monohydrate (AR, Sinopharm Chemical Reagent), and polyvinyl alcohol (PVA, AR, Sinopharm Chemical Reagent) were used without any further purification.

### Preparation of SFRuM perovskite

The perovskite powders were synthesized by a modified sol-gel method. For the synthesis of 0.03 mol (~12 g) of each perovskite, citric acid (15.0 g) and PVA (15.0 g) were added gradually in hot deionized water (70 °C, ~300 mL), metal precursors in the stoichiometric ratio were then dissolved to the above suspension and a clear solution precursor was obtained after concentration. A fluffy powder was obtained after the mild combustion on the heating plate, which was then calcined at 1100 °C for 10 h to obtain pure perovskite powder.

### Characterizations

In situ and ex situ XRD measurements were performed on PANalytical Empyrean diffractometer (Cu Kα radiation, λ = 1.5432 Å), and the spectra were collected in 2*θ* range of 20−80° at a scanning rate of 10° min^−1^. Rietveld refinement was performed using GSAS software, and the data were recorded in 2*θ* range of 10−110° with a scanning rate of 2° min^−1^. For in situ XRD measurements, the as-prepared SFRuM was initially heated to 800 °C, which was exposed to 50% H_2_/Ar (100 mL min^−1^) after Ar (50 mL min^−1^) purging to investigate the reduction process. The reoxidation process was conducted in the air after Ar (50 mL min^−1^) purging. This procedure was repeated twice under the same conditions.

XPS measurements were conducted with a spectrometer (PHOIBOS-100 analyzer from SPECS) equipped with an Al Kα X-ray source operated at 300 W. Sample preparation: ~300 mg perovskite powders were calcined in 5% H_2_/Ar (50 mL min^−1^) or air (50 mL min^−1^) in a tubular furnace at 800 °C for 2 h. A few milligrams of the reduced or reoxidized powders were collected during each process, which were pressed on the copper foam and transferred to the analysis chamber immediately.

In situ STEM measurements were performed on a HITACHI HF5000 (Environmental aberration-corrected TEM/STEM/SE) based on a standard 200 kV cold-field emission gun TEM, equipped with a highly sensitive secondary electron detector. Based on the surface and structural information from the SE, BF, and DF-STEM images, we can obtain a direct geometrical structure and acquire a deep understanding of the behavior under redox manipulations. In situ reduction and reoxidation of SFRuM were performed at 600−850 °C in 10 Pa H_2_ and O_2_ (2 mL min^−1^). In situ STEM-EDS elemental maps and STEM-EELS could be obtained simultaneously. Ex situ atomic-scale crystal structure and STEM-EDS elemental maps were also obtained using this instrument, and the specimen was prepared with Leica EM UC7 microtome.

Ex situ STEM-EDS elemental maps were also collected on JEOL JEM F200. SEM images were obtained on JSM-7800F. Raman spectrum was collected on a LabRAM HR 800 Raman spectrometer.

### Electrochemical measurements

SOEC with a configuration of SFRuM-GDC | LDC (La_0.4_Ce_0.6_O_2−*δ*_)|LSGM (fuel cell materials)|BSCF (Ba_0.5_Sr_0.5_Co_0.8_Fe_0.2_O_3−*δ*_)-GDC was fabricated. LSGM electrolyte was fabricated by dry-pressing LSGM powder under 200 MPa and then treated at 1450 °C for 10 h in air. The electrolyte pellet was ~500 µm thick and ~20 mm in diameter. BSCF was synthesized by a sol-gel method^31^. LDC and GDC fluorite phase powders were synthesized by a glycine combustion method^52^. The obtained LDC powder was mixed with viscid gum consisting of a-terpineol (Alfa Aesar), ethyl cellulose (SECOMA), and S-2800 (Shanghai Kaikai Chemical) with a mass ratio of 24:67:5:4 to prepare an LDC slurry by ultrasonic dispersion, which was spin-coated onto one side of the LSGM electrolyte and calcined at 1200 °C for 3 h to obtain LDC interlayer. SFRuM-GDC (60:40 wt%) composite cathode was assembled on the surface of the LDC side and BSCF-GDC (50:50 wt%) composite anode was assembled on the opposite side with an active area of 0.5 cm^2^, followed with treatment at 1100 °C for 2 h. CO_2_ electrolysis performance was measured on a homemade device^52^, Au paste was used as the current collector. The reduction and reoxidation processes of the cathode were conducted under 5% H_2_/Ar (20 mL min^−1^) and air (20 mL min^−1^) at 800 °C, respectively. 95% CO_2_ + 5% N_2_ was fed to the cathode (50 mL min^−1^, N_2_ as internal standard gas) and the products were detected by online gas chromatography (Agilent GC490). The measurements at different voltages were conducted using Metrohm Autolab potentiostat/galvanostat (PGSTAT 302 N). I–V curves were recorded from 0.3 to 1.6 V by a sequential step change of 10 mV s^−1^. EIS was recorded in a frequency range of 1 MHz to 0.1 Hz under an amplitude of 10 mV.

### Computational models and methods

Spin-polarized DFT calculations were implemented using a plane-wave basis set in the Vienna Ab-initio Simulation Packages (VASP 5.4)^53^. The exchange-correlation energy was treated using Perdew–Burke–Ernzerhof (PBE)^54^ functional within the generalized gradient approximation (GGA)^55^. The projected-augmented wave (PAW)^56^ pseudopotentials were utilized to describe the core electrons, and a cutoff energy of 400 eV was used for the plane-wave expansion. The following valence electron configurations were included in the self-consistent field calculations: Sr (4*s*^2^, 4*p*^6^, and 5*s*^2^), Fe (3*d*^6^ and 4*s*^2^), Mo (4*d*^5^ and 5*s*^1^), Ru (4*d*^7^ and 5*s*^1^), O (2**s**^2^ and 2*p*^4^), and C (2*s*^2^ and 2*p*^2^). In addition, the van der Waals (vdW) dispersion forces were corrected by the vdW-DF (optPBE) function, which showed a highly accurate description for oxides^57^. An on-site Hubbard term U-J was added to address the open-shell *d*-electrons (2.89 eV for Fe)^57^. The energies and residual forces were converged to 10^−6^ eV and 0.01 eV Å^−1^, respectively.

### Models

According to the characterizations by XRD and TEM in Supplementary Fig. 1, the crystal structures are referenced to SFM (Sr_2_Fe_1.5_Mo_0.5_O_6−*δ*_) (*Pnma*, no. 62) before reduction and RP-SFM (Sr_3_FeMoO_7−*δ*_) (*I*4*/mmm*, no. 139) after reduction. The optimal lattice constants are *a* = *c* = 5.50 Å and *b* = 8.00 Å for a ferromagnetic (FiM) Sr_4_Fe_3_MoO_12_ (SFM) cell, and *a* = *b* = 3.93 Å and *c* = 20.84 Å for a FiM Sr_6_Fe_3_MoO_14_ (RP-SFM) cell (Supplementary Fig. 32)^31^. Accordingly, a Sr_18_Fe_8_Mo_4_O_42_ supercell was adopted to warrant the stoichiometry ratio of Fe:Mo = 2:1. Here, we referred to SFM and RP-SFM as SFRuM and RP-SFRuM with consideration of only trace Ru in bulk. For structural evolution, the SFRuM[010] orientation is considered to explain the segregation ability of B-site dopants with or without oxygen vacancy and/or exsolved Ru. A slab with four Fe-O and three Sr-O layers was utilized to mimic the SFRuM(010) facet, where the bottom two layers were constrained. To evaluate the catalytic performance, the SFRuM(121) facet were used to model the pristine surface, where three Sr-Fe-O layers was included with the bottom two fixed (Supplementary Fig. 33a). And a simplified RP-SFRuM(105) ribbon integrated with a Fe_3_Ru_3_ cluster was utilized to simulate the interface structure, whose bottom two layers were constrained (RuFe@SFRuM, Supplementary Fig. 33b, c). For irreducible Brillouin zone sampling, a Monkhorst-Pack^58^ k-point grid of 4 × 4 × 4 was adopted for Sr_4_Fe_3_MoO_12_, 6 × 6 × 1 for Sr_6_Fe_3_MoO_14_, and 6 × 2 × 1 for Sr_18_Fe_8_Mo_4_O_42_. A gamma point was exploited for all the surface and interface models. Denser k-point grids were adopted for the PDOS calculation. The vacuum thickness was more than 12 Å between slabs.

### Thermodynamic corrections and formulae

Energy levels of the states were corrected to Gibbs free energies by the formula:

1 $${G\left(T\right)=E}_{{{{{{\rm{elec}}}}}}}+{{{{{{\mathrm{ZPE}}}}}}}+{{\delta }}H-T\Delta S$$

Where ${E}_{{{{{{\rm{elec}}}}}}}$ is the electronic energy calculated by DFT at 0 K, ${ZPE}$ is the zero-point energy, ${{\delta }}H$ is the integral of heat capacity, and $T\Delta S$ is the energy of entropy change (*T* = 800 °C). For adsorbed species, the last three items were obtained by vibrational frequencies calculations via standard methods^59^. For gaseous species, the corrections were taken from the *NIST* database through standard ideal-gas methods^60^. The relevant values are listed in Supplementary Table 4.

The exchange energy^28^ (${E}_{{{{{{\rm{exc}}}}}}}$) and segregation energy^14,35^ (${E}_{{{{{{\rm{seg}}}}}}}$) of B-site metals (X) were defined as:

2 $${E}_{{{{{\rm{exc}}}}}}={E}_{{{{{{\rm{X}}}}}}\_{{{{{\rm{nL}}}}}}}-{E}_{{{{{{\rm{X}}}}}}\_3{{{{{\rm{L}}}}}}}$$

3 $${E}_{{{{{{\rm{seg}}}}}}}={E}_{{\left({{{{{\rm{X}}}}}}\right)}_{{{{{{\rm{surf}}}}}}}}-{E}_{{\left({{{{{\rm{X}}}}}}\right)}_{{{{{{\rm{bulk}}}}}}}}$$

Where ${E}_{{{{{{\rm{X}}}}}}{\_nL}}$ is the energy of locating X at the *n*-th layer away from the surface layer (the first layer is termed 1L, henceforth). The third layer (3L) is treated as the bulk location, which energy is ${E}_{{{{{{\rm{X}}}}}}\_3{{{{{\rm{L}}}}}}}$ as the zero reference. ${E}_{\left({{{{{\rm{X}}}}}}\right)\_{{{{{\rm{surf}}}}}}}$ and ${E}_{\left({{{{{\rm{X}}}}}}\right)\_{{{{{\rm{bulk}}}}}}}$ are the total energies of the systems with X located at the surface and in the bulk, respectively. Here, a negative value means high stability.

The adsorption energy (${E}_{{{{{{\rm{ads}}}}}}\_C{{{{{{\rm{O}}}}}}}_{2}}$) of CO_2_ were calculated by:

4 $${E}_{{{{{{\rm{ads}}}}}}\_C{{{{{{\rm{O}}}}}}}_{2}}={E}_{{{{{{\rm{C}}}}}}{{{{{{\rm{O}}}}}}}_{2}\ast }-{E}_{\ast }-{E}_{{{{{{\rm{C}}}}}}{{{{{{\rm{O}}}}}}}_{2}}$$

Where ${E}_{{{{{{\rm{C}}}}}}{{{{{{\rm{O}}}}}}}_{2}\ast }$ is the energy of a CO_2_ adsorbed on the site (*), ${{{{{{\rm{E}}}}}}}_{\ast }$ is the energy of a clean site, and ${E}_{{{{{{\rm{C}}}}}}{{{{{{\rm{O}}}}}}}_{2}}$ is the energy of the gaseous CO_2_.

The formation energy of oxygen vacancy (*E*_ovf_) is defined as:

5 $${E}_{{{{{{\rm{ovf}}}}}}}={E}_{{{{{{{\rm{V}}}}}}}_{{{{{{\rm{O}}}}}}}^{\cdot \cdot }}+{E}_{{{{{{{\rm{CO}}}}}}}_{2}}-{E}_{{{{{{\rm{int}}}}}}}-{E}_{{{{{{\rm{CO}}}}}}}$$

Where ${E}_{{{{{{{\rm{V}}}}}}}_{{{{{{\rm{O}}}}}}}^{\cdot \cdot }}$ and ${E}_{{{{{{\rm{int}}}}}}}$ are the total energies of the oxygen-defected and the intact surfaces. ${E}_{{{{{{\rm{CO}}}}}}}$ is the energy of gaseous CO.

The electron density differences were calculated using the formula:

6 $$\Delta \rho ={\rho }_{{{{{{\rm{C}}}}}}{{{{{{\rm{O}}}}}}}_{2}\ast ({{{{{{\rm{V}}}}}}}_{{{{{{\rm{O}}}}}}}^{\cdot \cdot })}-{\rho }_{\ast ({{{{{{\rm{V}}}}}}}_{{{{{{\rm{O}}}}}}}^{\cdot \cdot })}-{\rho }_{{{{{{\rm{C}}}}}}{{{{{{\rm{O}}}}}}}_{2}}$$

## Supplementary information

Supplementary Information

Peer Review File

Description of Additional Supplementary Files

Supplementary Movie 1

Supplementary Movie 2

## Data Availability

The data that support the findings of this study are available from the corresponding author upon reasonable request due to the data are of large amount. Source data are provided with this paper.

## Source data

Source Data
