# Supplementary material for: Promoting exsolution of RuFe alloy nanoparticles on Sr2Fe1.4Ru0.1Mo0.5O6−δ via repeated redox manipulations for CO2 electrolysis
Source: Nat Commun. 2021 Sep 27;12:5665. doi: 10.1038/s41467-021-26001-8 (PMC8476569; doi:10.1038/s41467-021-26001-8)
Supplement: Supplementary file 1 — Supplementary Information [file 41467_2021_26001_MOESM1_ESM.pdf]

Promoting exsolution of RuFe alloy nanoparticles on $Sr_2Fe_{1.4}Ru_{0.1}Mo_{0.5}O_{6-\delta}$ via repeated redox manipulations for CO_2 electrolysis

Houfu Lv[1,2,†], Le Lin[1,3,†], Xiaomin Zhang[1], Rongtan Li[1,2], Yuefeng Song[1], Hiroaki Matsumoto[4], Na Ta[1], Chaobin Zeng[4], Qiang Fu[1], Guoxiong Wang[1]* and Xinhe Bao[1]

[1]State Key Laboratory of Catalysis, Dalian National Laboratory for Clean Energy, Dalian Institute of Chemical Physics, Chinese Academy of Sciences, Dalian, P. R. China

[2]University of Chinese Academy of Sciences, Beijing, P. R. China

[3]School of Physical Science and Technology, ShanghaiTech University, Shanghai, P. R. China

[4]Hitachi High-tech (Shanghai) Co., Ltd., Shanghai, P. R. China

[†]These authors contributed equally to this work.

E-mail: wanggx@dicp.ac.cn

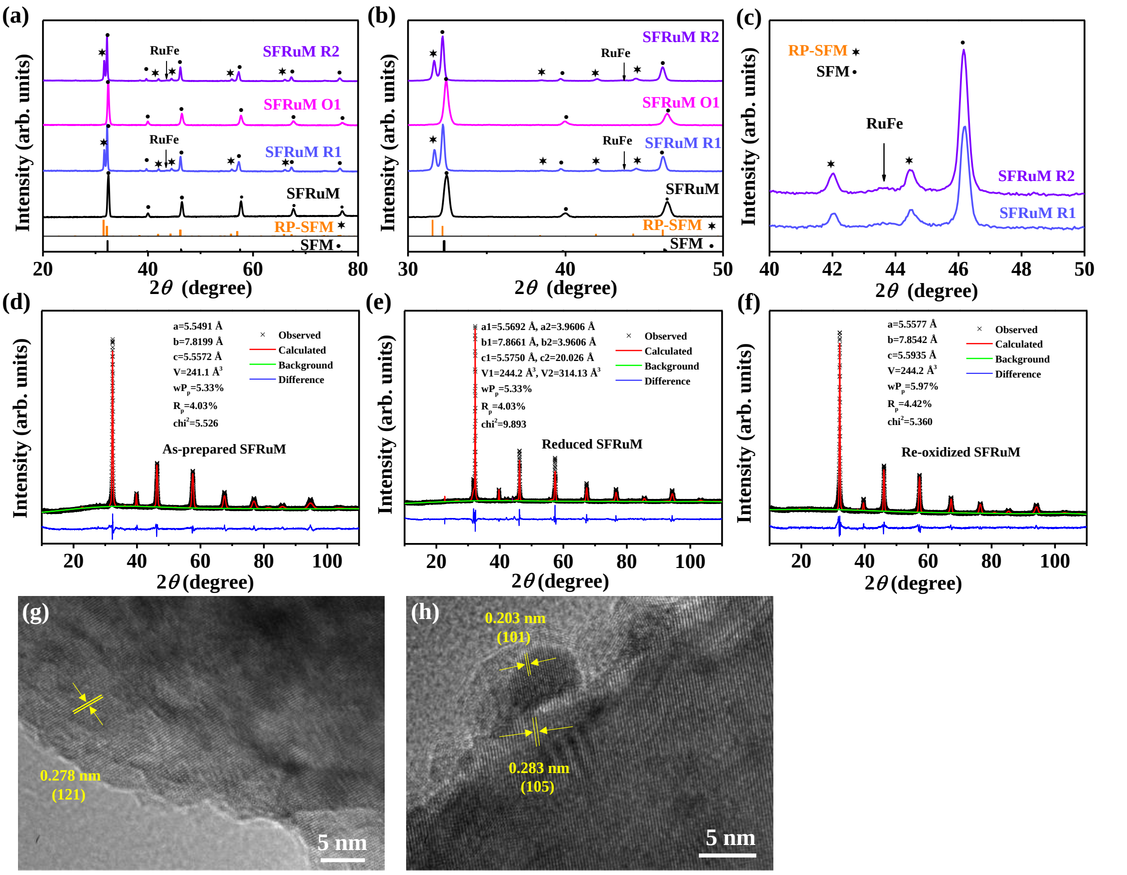

Supplementary Figure 1. (**a**) Ex situ XRD patterns of as-prepared SFRuM, reduced SFRuM in 5% H_2/Ar supplied at 800 ℃ for 2 h (SFRuM R1), re-oxidized SFRuM R1 in air supplied at 800 ℃ for 2 h (SFRuM O1), and re-reduced SFRuM O1 in in 5% H_2/Ar supplied at 800 ℃ for 2 h (SFRuM R2). (**b**) The enlarged XRD patterns at 30-50 ° of (**a**). (**c**) The enlarged XRD patterns of SFRuM R1 and SFRuM R2 at 40-50 °. XRD Rietveld refinement patterns of as-prepared (**d**), reduced (**e**) and re-oxidized (**f**) SFRuM powders. (**g**) HRTEM image of the as-prepared SFRuM. (**h**) HRTEM image of a typical RuFe alloy nanoparticle (NP) and the RP-SFRuM.

High resolution transmission electron microscopy (HRTEM) image of as-prepared $Sr_2Fe_{1.4}Ru_{0.1}Mo_{0.5}O_{6-\delta}$ (SFRuM) in (c) demonstrates a lattice parameter of 0.278 nm, corresponding to the (121) plane of the double perovskite (PDF#01-080-8394). The lattice parameters of 0.203 nm and 0.283 nm after reduction in (d), corresponding to the (101) plane of RuFe alloy phase and (105) plane of Ruddlesden-Popper phase $Sr_3Fe_{2\text{-}x\text{-}y}Ru_yMo_xO_{7-\delta}$ (RP-SFRuM, PDF#01-075-3655), respectively. The exsolution process is shown below:

$$Sr_2Fe_{1.4}Ru_{0.1}Mo_{0.5}O_{6-\delta} + H_2 \rightarrow Sr_3Fe_{2-x-y}Ru_yMo_xO_{7-\delta} + Sr_2(Fe_{1.4}Ru_{0.1}Mo_{0.5})_{1-z}O_{6\text{-}\delta} + RuFe + H_2O + V_O^{\cdot\cdot}$$

Where, $Sr_2(Fe_{1.4}Ru_{0.1}Mo_{0.5})_{1-z}O_{6-\delta}$ represents B-site deficient double perovskite after reduction and before the phase transformation. $V_O^{\cdot\cdot}$ represents the oxygen vacancy.

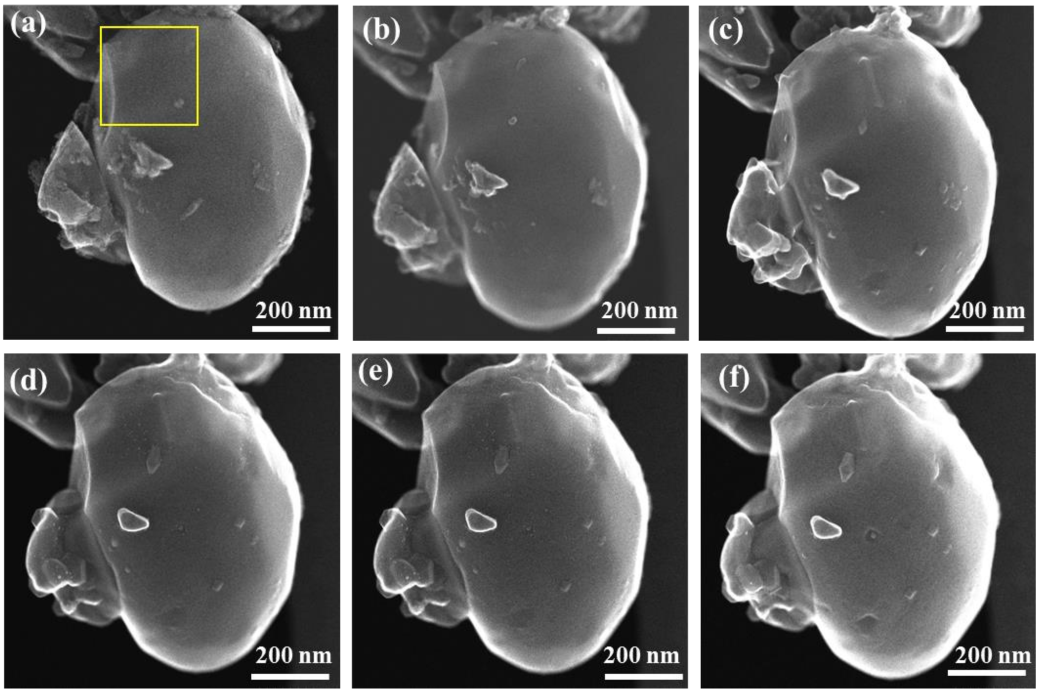

Supplementary Figure 2. In situ scanning transmission electron microscopy (STEM) results, secondary electron (SE)-STEM images of as-prepared SFRuM. (**a**) Before reduction. (**b**) After reduction at 600 ℃ for ~10 min. (**c**) After reduction at 800 ℃ for ~15 min. (**d**) After reduction at 800 ℃ for ~30 min. (**e**) After re-oxidation at 800 ℃ for ~30 min. (**f**) After re-oxidation in 10 Pa of O_2 supplied at 800 ℃ for ~40 min.

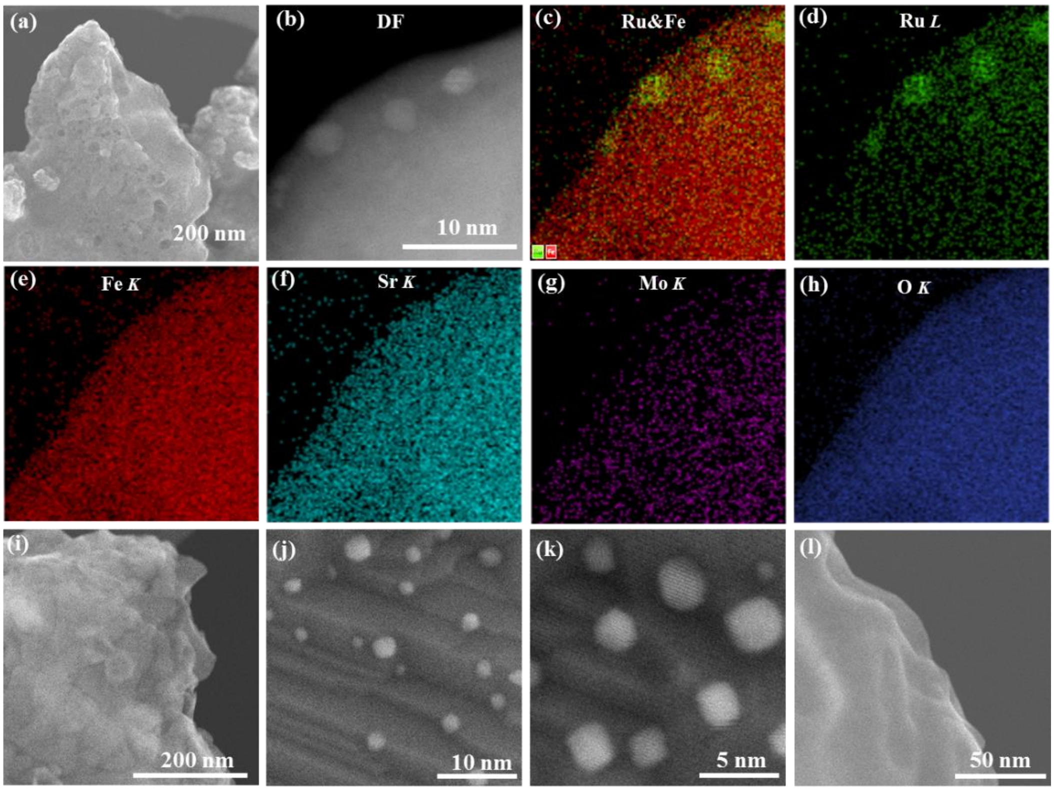

Supplementary Figure 3. In situ STEM results of SFRuM O1. (**a**) SE-STEM image of SFRuM O1 before reduction in Fig. 1e,f. (**b**) DF-STEM image of SFRuM O1 after reduction at 600 ℃ for ~10 min. (**c-h**) The corresponding STEM-EDS elemental maps of (**b**). SE-STEM image of SFRuM O1 before reduction (**i**), after reduction at 800 ℃ for ~60 min (**j**,**k**), and after re-oxidation at 800 ℃ for ~60 min (**l**) in Fig. 1g,h.

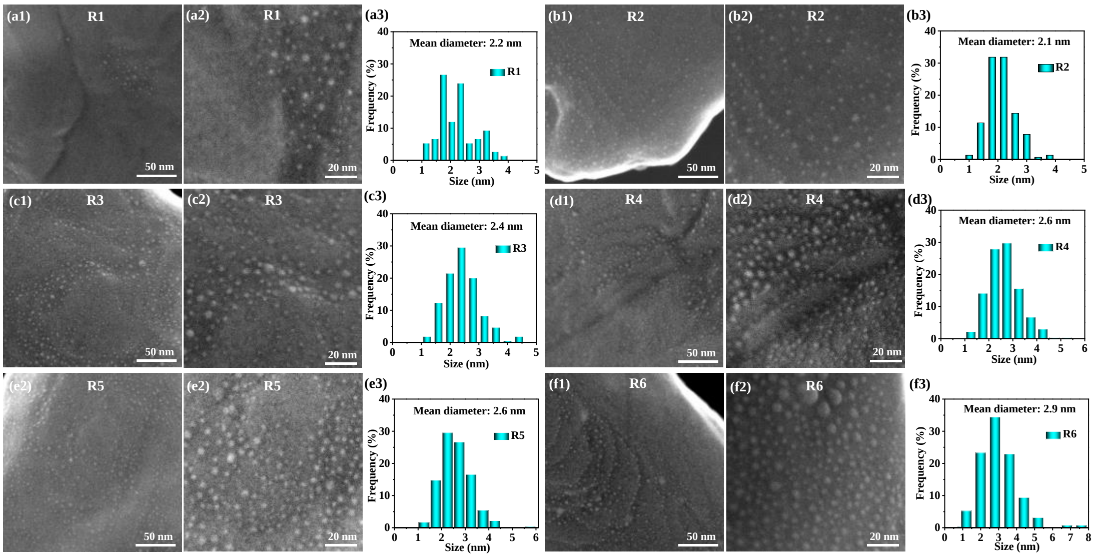

Supplementary Figure 4. Ex situ SE-STEM images, particle population and size of the exsolved NPs on SFRuM after different reduction processes in redox manipulations. (**a**) SFRuM R1. (**b**) SFRuM R2. (**c**) SFRuM R3. (**d**) SFRuM R4. (**e**) SFRuM R5. (**f**) SFRuM R6. The particle population in (**a2**), (**b2**), (**c2**), (**d2**), (**e2**) and (**f2**) is 75, 154, 220, 269, 278 and 288, respectively.

SFRuM $\xrightarrow{800\ ^{o}C\ 5\%\ H_2\ 2h}$ SFRuM R1 $\xrightarrow{800\ ^{o}C\ Air\ 2h}$ SFRuM O1

SFRuM O1 $\xrightarrow{800\ ^{o}C\ 5\%\ H_2\ 2h}$ SFRuM R2 $\xrightarrow{800\ ^{o}C\ Air\ 2h}$ SFRuM O2

SFRuM O2 $\xrightarrow{800\ ^{o}C\ 5\%\ H_2\ 2h}$ SFRuM R3 $\xrightarrow{800\ ^{o}C\ Air\ 2h}$ SFRuM O3

SFRuM O3 $\xrightarrow{800\ ^{o}C\ 5\%\ H_2\ 2h}$ SFRuM R4 $\xrightarrow{800\ ^{o}C\ Air\ 2h}$ SFRuM O4

SFRuM O4 $\xrightarrow{800\ ^{o}C\ 5\%\ H_2\ 2h}$ SFRuM R5 $\xrightarrow{800\ ^{o}C\ Air\ 2h}$ SFRuM O5

SFRuM O5 $\xrightarrow{800\ ^{o}C\ 5\%\ H_2\ 2h}$ SFRuM R6 $\xrightarrow{800\ ^{o}C\ Air\ 2h}$ SFRuM O6

As-prepared SFRuM shows glossy particle surfaces and a few metal NPs were distributed on the surface of SFRuM after the first reduction (SFRuM R1, (a)). After re-oxidation, a complete dissolution of the NPs into the perovskite occurred. After the second reduction process (SFRuM R2), more ultrafine NPs were exsolved (b). Much more NPs could be exsolved on SFRuM R3 (c). The relationship between the density of the exsolved NPs and the number of the redox cycles is shown in Fig. 1l, and the population of exsolved NPs increases with the number of redox cycles. In consequence, exsolution is greatly promoted through repeated redox manipulations, which is probably due to Ru enrichment underneath the SFRuM surface.

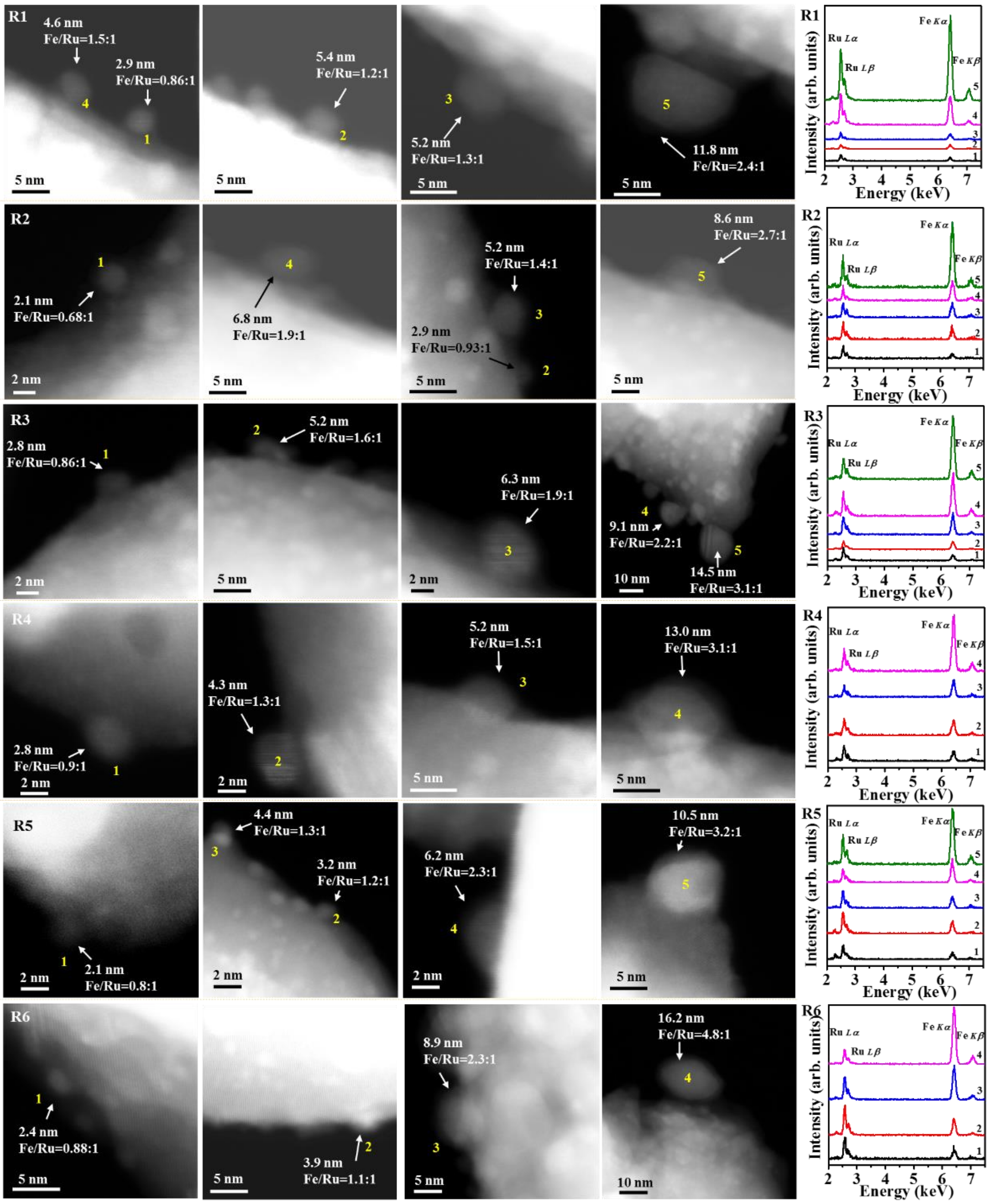

Supplementary Figure 5. DF-STEM images and the corresponding STEM-EDS results in SFRuM after different redox manipulations.

The atomic ratio of Fe/Ru in the RuFe alloy NPs varies with the size of the exsolved NPs, which has no concern with the number of redox manipulations. The ratio of Fe/Ru is around 0.7:1~1.1:1, 1:1~1.6:1, 1.9:1~2.7:1, and >2.4:1 when the size of the NPs are 1~3 nm, 3~6 nm, 6~10 nm, and >10 nm, respectively. The mean size of the exsolved NPs is 2.8 nm (Fig. 1l), suggesting that the metal/perovskite interfaces with a Fe/Ru ratio of 0.7:1~1.1:1 in the RuFe alloy NPs dominate the catalytic activity.

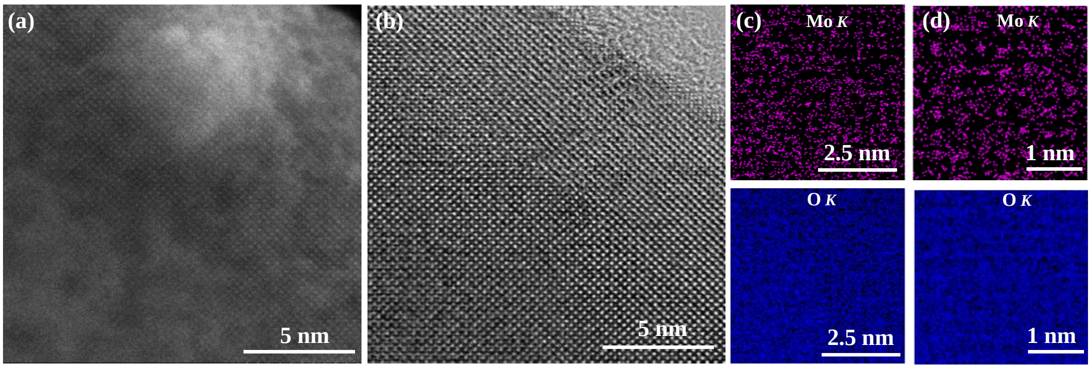

Supplementary Figure 6. Atomic-scale STEM images of SFRuM R1. (**a**) SE-STEM image. (**b**) Bright field (BF)-STEM image. (**c**, **d**) Atomic-scale EDS elemental maps of Fig. 2b,c.

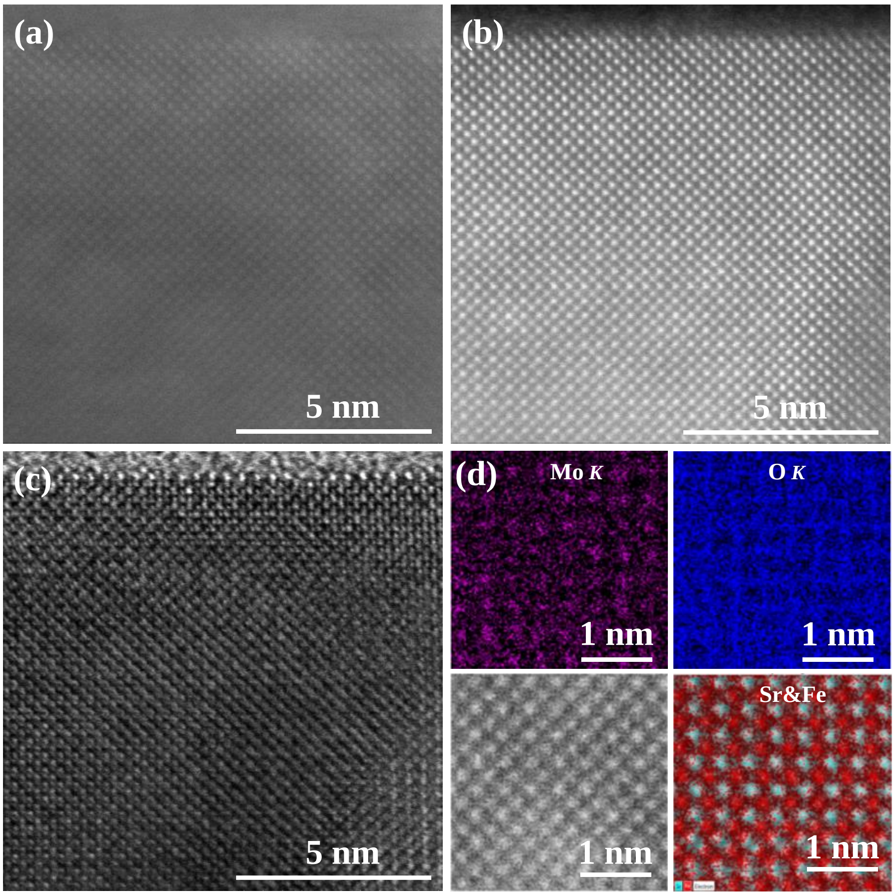

Supplementary Figure 7. Atomic-scale STEM images of SFRuM O1. (**a**) SE-STEM image. (**b**) High angle annular dark field (HAADF)-STEM image. (**c**) BF-STEM image. (**d**) Atomic-scale STEM-EDS elemental maps of Fig. 2d.

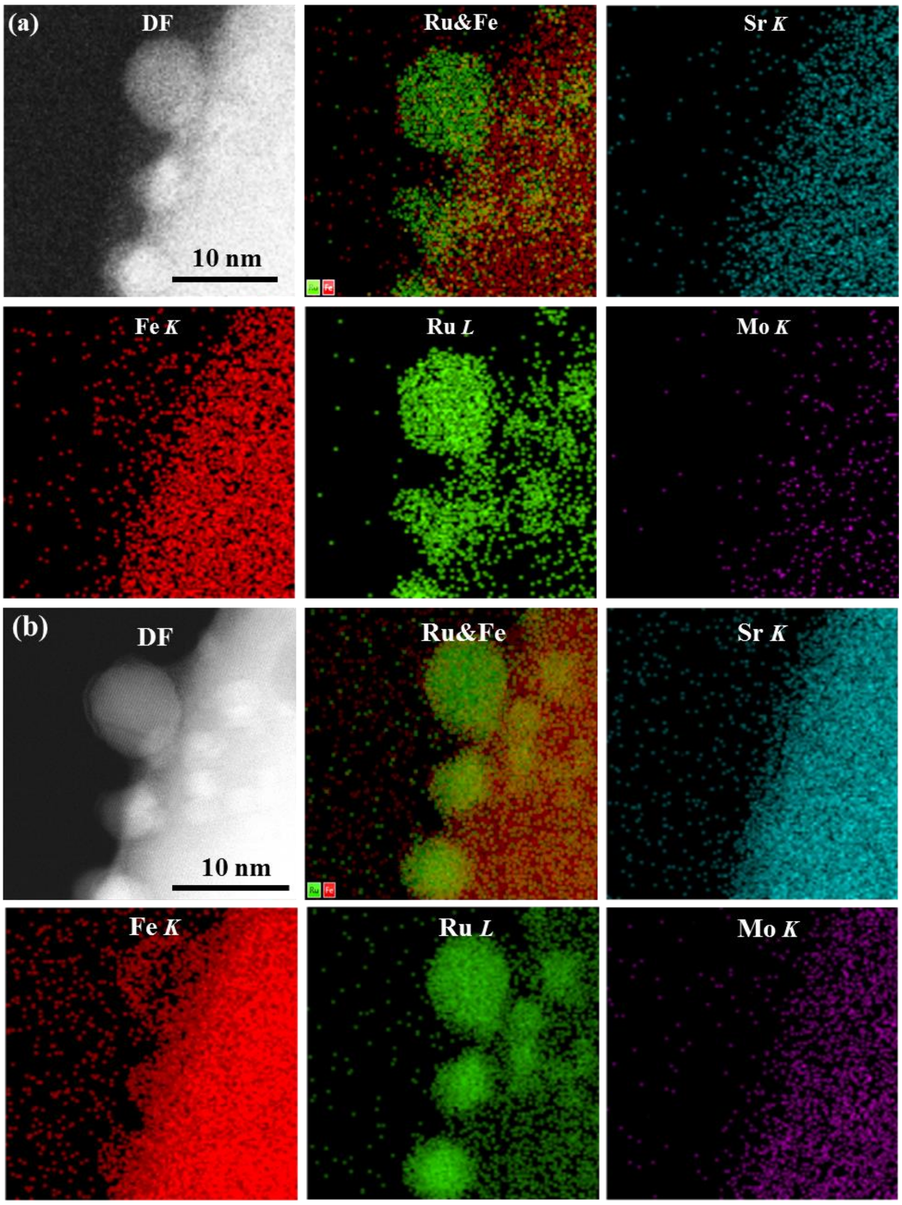

Supplementary Figure 8. In situ DF-STEM image and the corresponding STEM-EDS elemental maps of SFRuM O1 after reduction at 800 ℃ for ~15 min (**a**), and after reduction at 850 ℃ for another ~15 min (**b**).

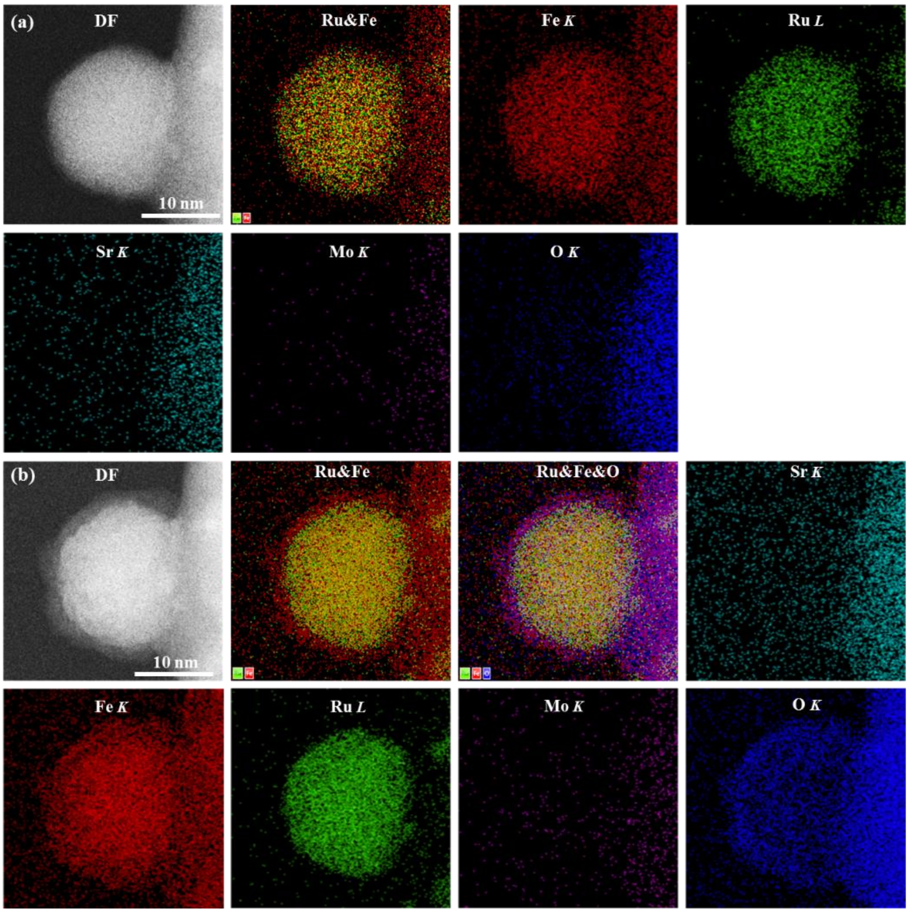

Supplementary Figure 9. In situ STEM and STEM-EDS results. (**a**,**b**) DF-STEM images and STEM-EDS elemental maps of Fig. 2j and k.

Uniform RuFe alloy NPs can be obtained after reduction at 800 ℃ for ~60 min and then at 850 ℃ for ~30 min (Supplementary Fig. 9a). After re-oxidation in 10 Pa of O_2 at 200 ℃ for ~70 s, a FeO_x shell could be obtained (Supplementary Fig. 9b).

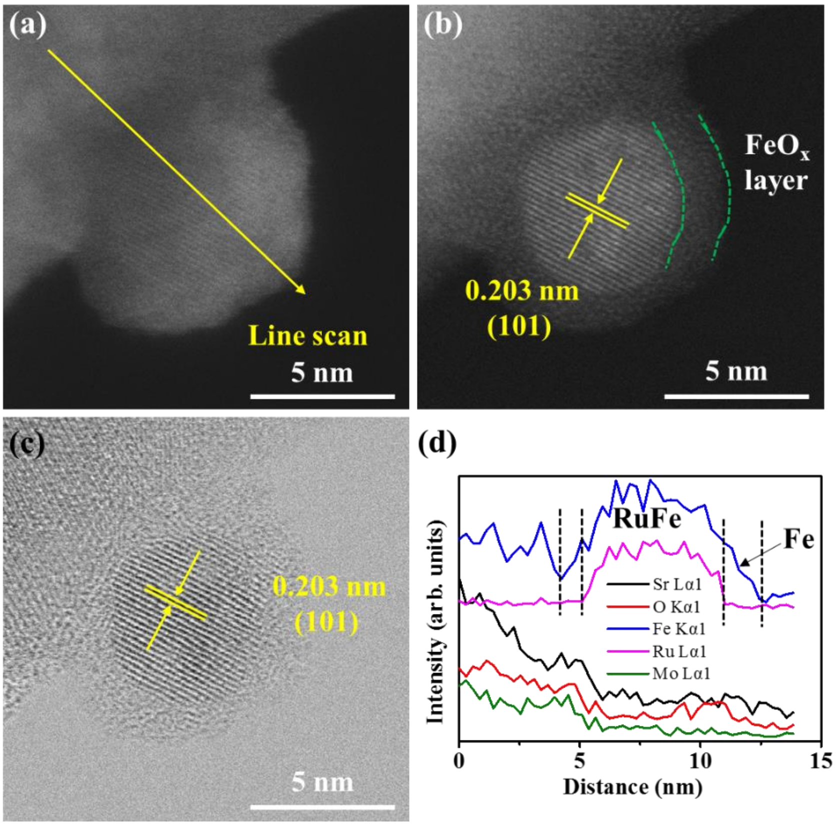

Supplementary Figure 10. STEM results of SFRuM R1. (**a**) SE-STEM image. (**b**) DF-STEM image. (**c**) BF-STEM image. (**d**) STEM line scan result in (**a**).

In Supplementary Fig. 10, a decoration of the NP with amorphous layer of FeO_x with low contrast on the surface was observed.

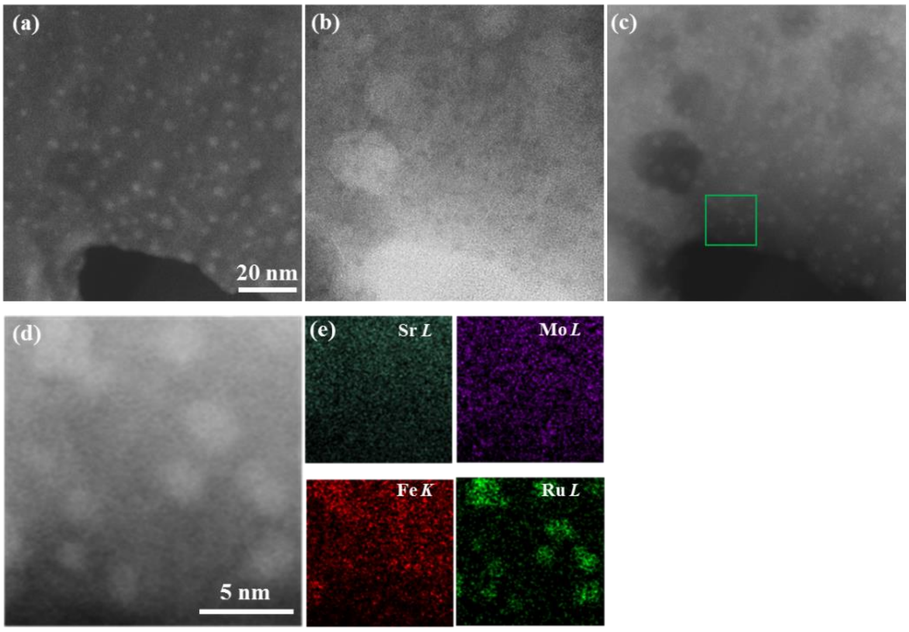

Supplementary Figure 11. STEM results of SFRuM R3. (**a**) SE-STEM image. (**b**) BF-STEM image. (**c**) DF-STEM image. (**d**) Enlarged DF-STEM image in (**c**). (**e**) STEM-EDS elemental maps of (**d**).

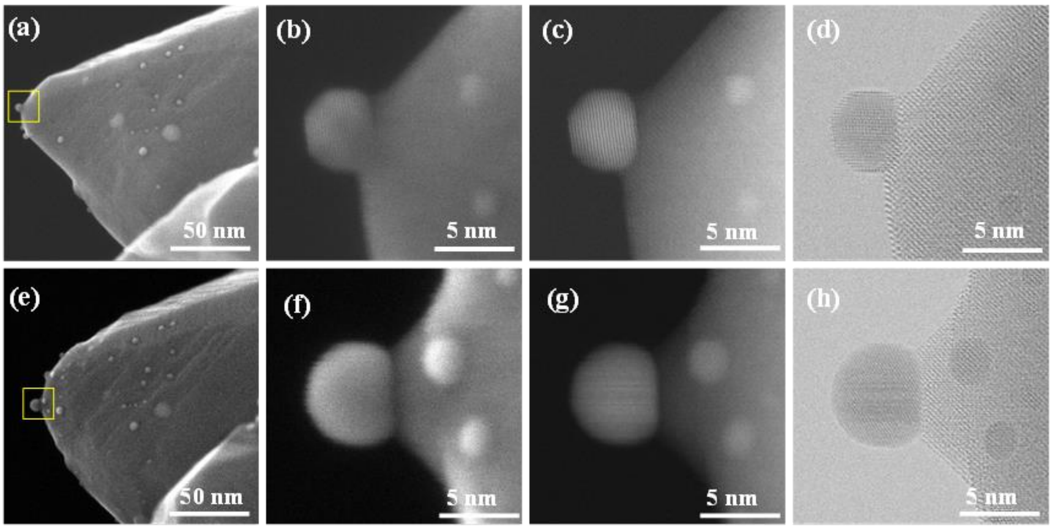

Supplementary Figure 12. In situ STEM results of SFRuM O1. (**a**) After reduction in 10 Pa of H_2 supplied at 800 ℃ for ~15 min. Partially enlarged images, (**b**) SE-STEM image, (**c**) DF-STEM image and (**d**) BF-STEM image. (**e**) After reduction in 10 Pa of H_2 supplied at 850 ℃ for another ~15 min. Partially enlarged images, (**f**) SE-STEM image, (**g**) DF-STEM image and (**h**) BF-STEM image.

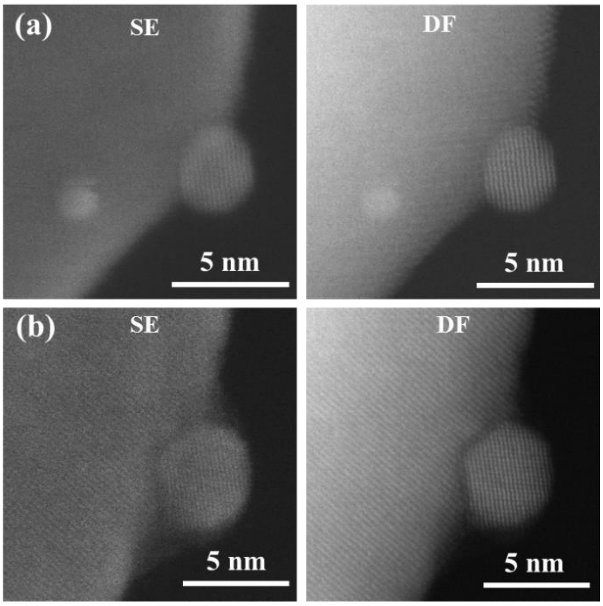

Supplementary Figure 13. In situ SE-STEM and DF-STEM images of Fig. 2h (**a**) and Fig. 2i (**b**).

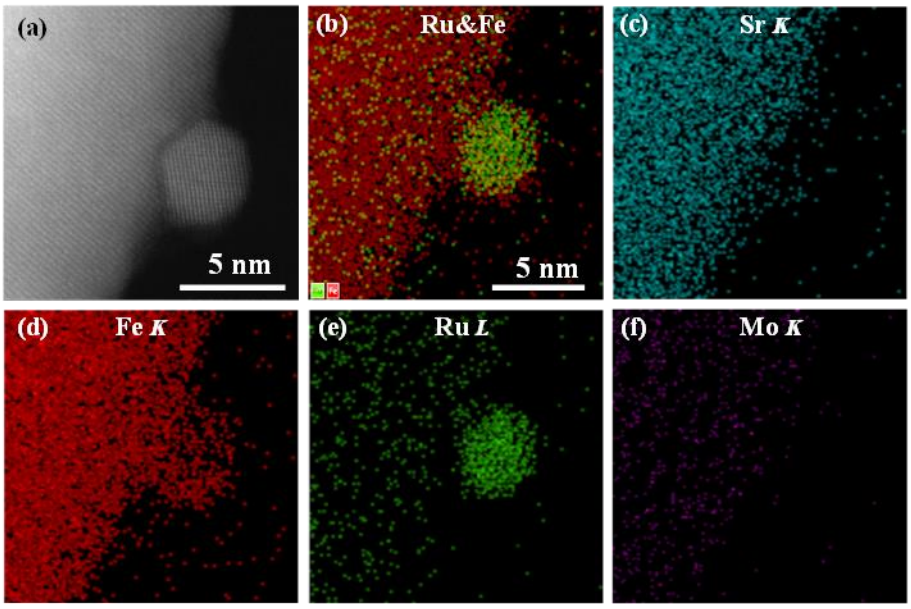

Supplementary Figure 14. In situ STEM-EDS results of Fig. 2i. (**a**) DF-STEM image. (**b**-**f**) STEM-EDS elemental maps of overlapped Ru&Fe, Sr, Fe, Ru, and Mo.

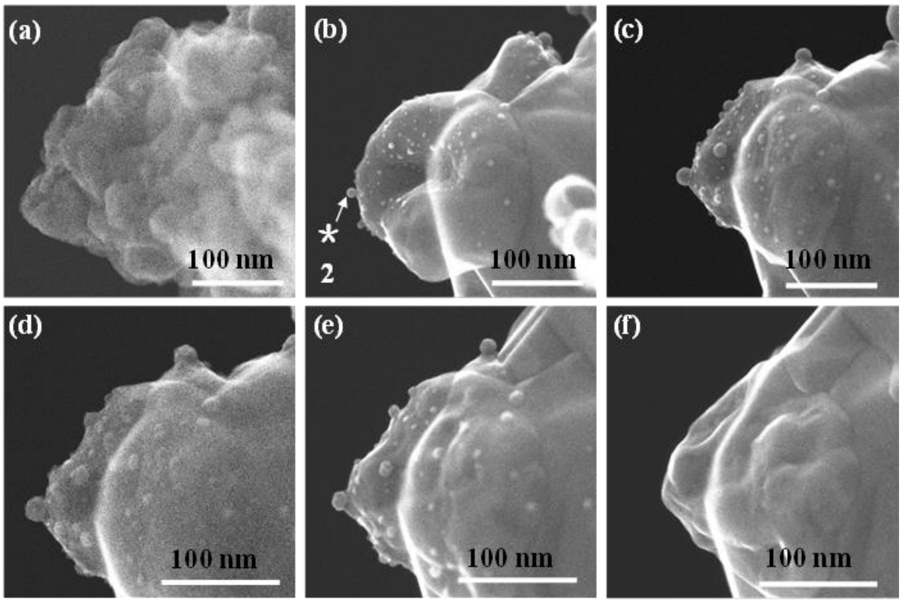

Supplementary Figure 15. In situ STEM results. SE-STEM images of SFRuM O1 before reduction (**a**), after reduction in 10 Pa of H_2 supplied at 800 ℃ for ~60 min (**b**), after reduction in 10 Pa of H_2 supplied at 850 ℃ for another ~30 min (**c**), after re-oxidation in 10 Pa of O_2 supplied at 200 ℃ for ~70 s (**d**), after re-oxidation in 10 Pa of O_2 supplied at 800 ℃ for ~30 min (**e**) and after re-oxidation in 10 Pa of O_2 supplied at 800 ℃ for ~60 min (**f**).

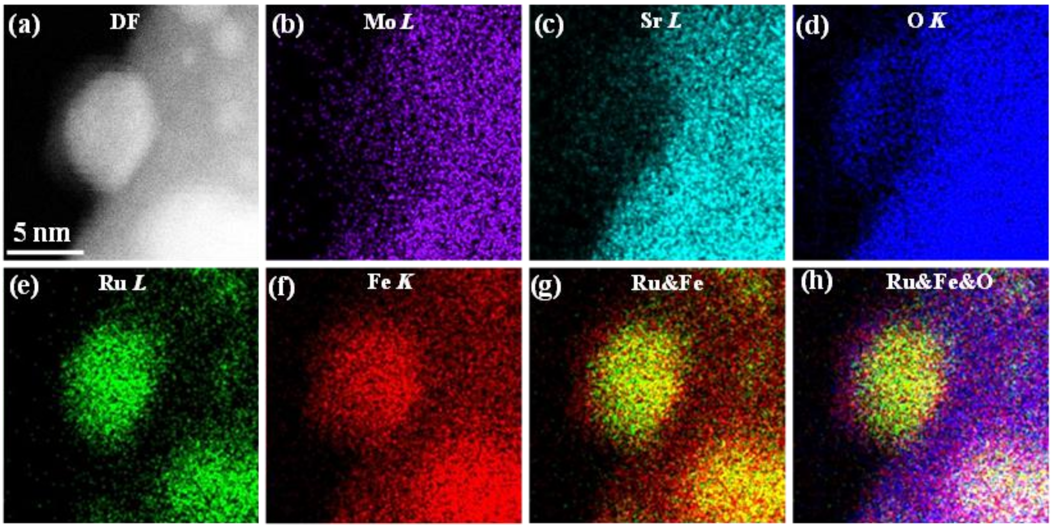

Supplementary Figure 16. STEM-EDS results of SFRuM R1 (after exposure to air for ~3 min). (**a**) DF-STEM image. (**b**-**h**) STEM-EDS elemental maps of Sr, Fe, Ru, Mo, O, overlapped Ru&Fe, and overlapped Ru&Fe&O of (**a**).

The sample was reduced under 5% H_2/Ar (50 mL min^{-1}) in a tubular furnace at 800 ℃ for 2 h and transferred to the STEM chamber immediately to avoid the environmental impacts, but an oxide layer could also be seen (~0.5 nm) even for a very short time (~3 min) in air at room temperature. During the O_2 annealing, Fe, with a strong O_2 affinity, diffuses out onto the surface of RuFe alloy NPs, forming a thin FeO_x coating, similar to oxidation-induced segregation of transition metals as reported in the FePt[1], FeNi[2], and FePd[3] systems.

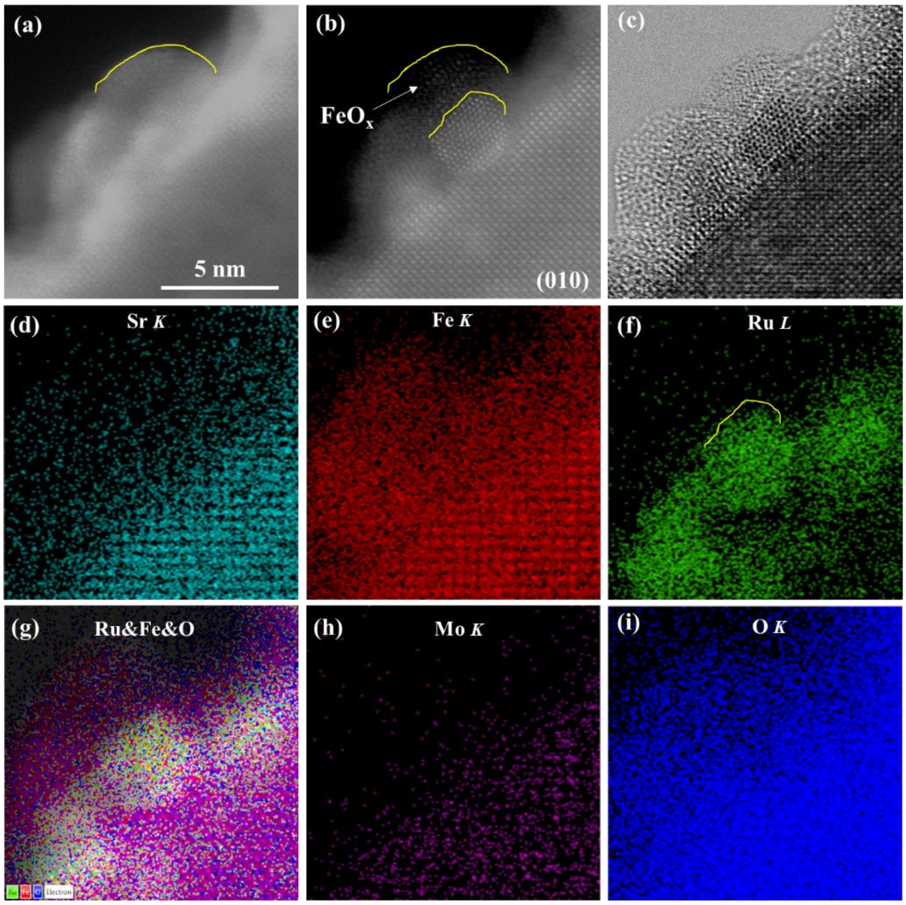

Supplementary Figure 17. STEM-EDS results of SFRuM R1 (after exposure to air for ~15 days). (**a**) SE-STEM image. (**b**) HAADF-STEM image. (**c**) BF-STEM image. (**d-i**) STEM-EDS elemental maps of Sr, Fe, Ru, Mo, O, and overlapped Ru&Fe&O.

The specimen was prepared with Leica microtome to obtain the thin region, and the oxide layer is significantly thickened with prolonged time in air.

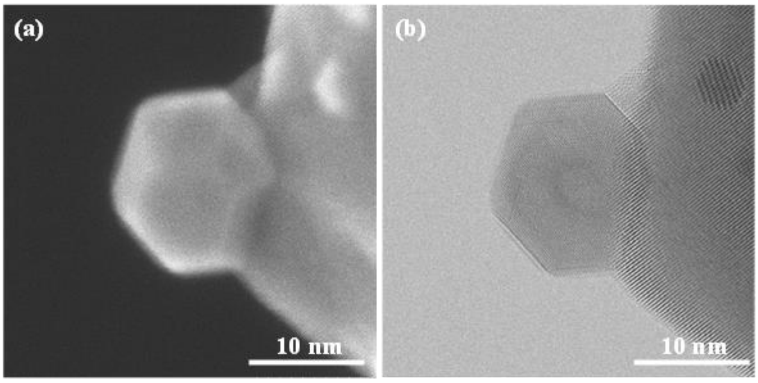

Supplementary Figure 18. In situ STEM results. (**a**) SE-STEM and (**b**) BF-STEM images of Fig. 2l.

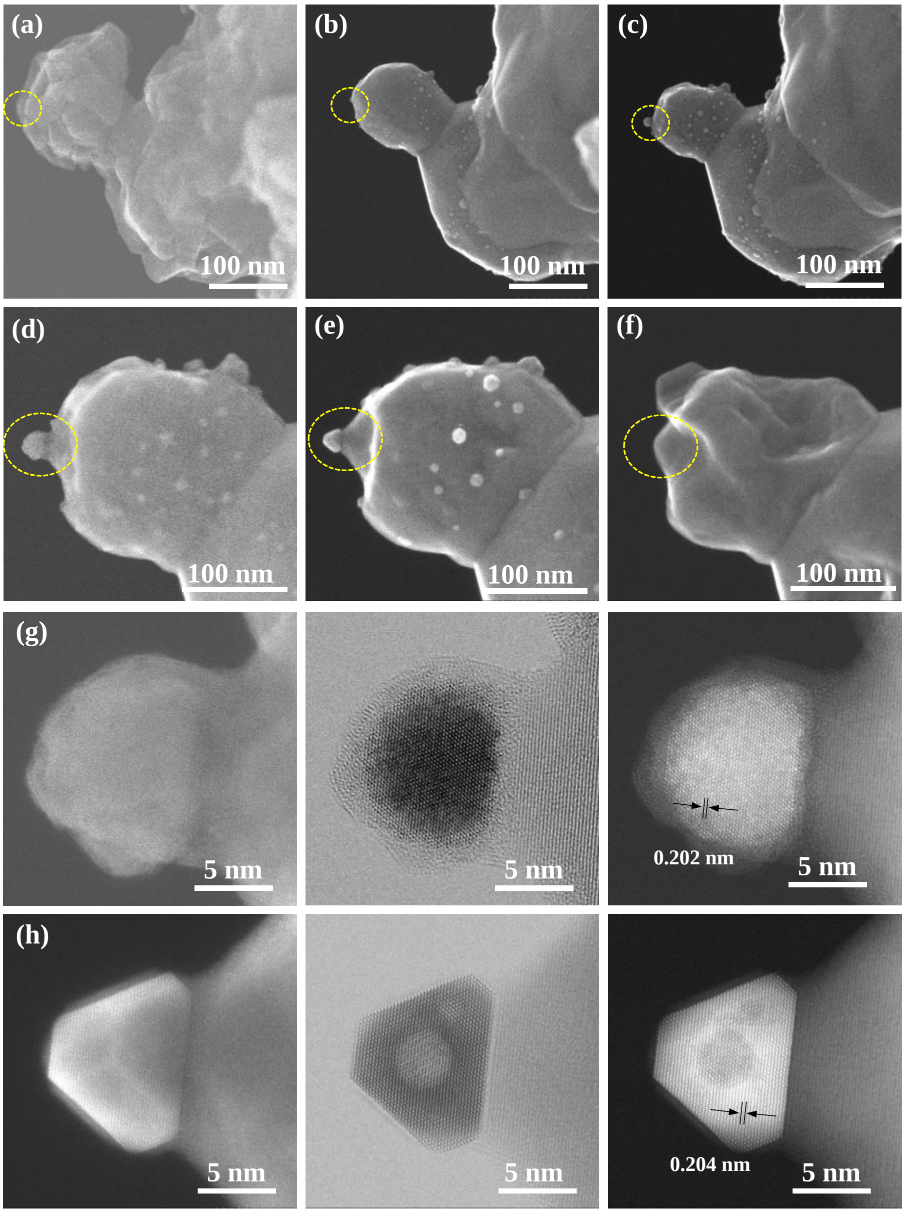

Supplementary Figure 19. In situ STEM results. SE-STEM images of SFRuM O1 before reduction (**a**), after reduction at 800 ℃ for ~60 min (**b**), after reduction at 850 ℃ for another ~30 min (**c**), after re-oxidation at 200 ℃ for ~70 s (**d**), after re-oxidation at 800 ℃ for ~30 min (**e**) and after re-oxidation at 800 ℃ for ~60 min (**f**). (**g**) SE, BF and DF-STEM images of a typical particle in (**d**). (**h**) SE, BF and DF-STEM images of a typical particle in (**e**).

RuFe alloy NPs emerged after reduction and vanished after re-oxidation. After in situ re-oxidation at 200 ℃, Fe, with a strong O_2 affinity, was oxidized to the surface of RuFe alloy NPs, forming a thin FeO_x overlayer, which dissolved back into the perovskite preferentially, leaving a metallic Ru-enriched NP. Subsequently, all the NPs dissolved into the perovskite completely.

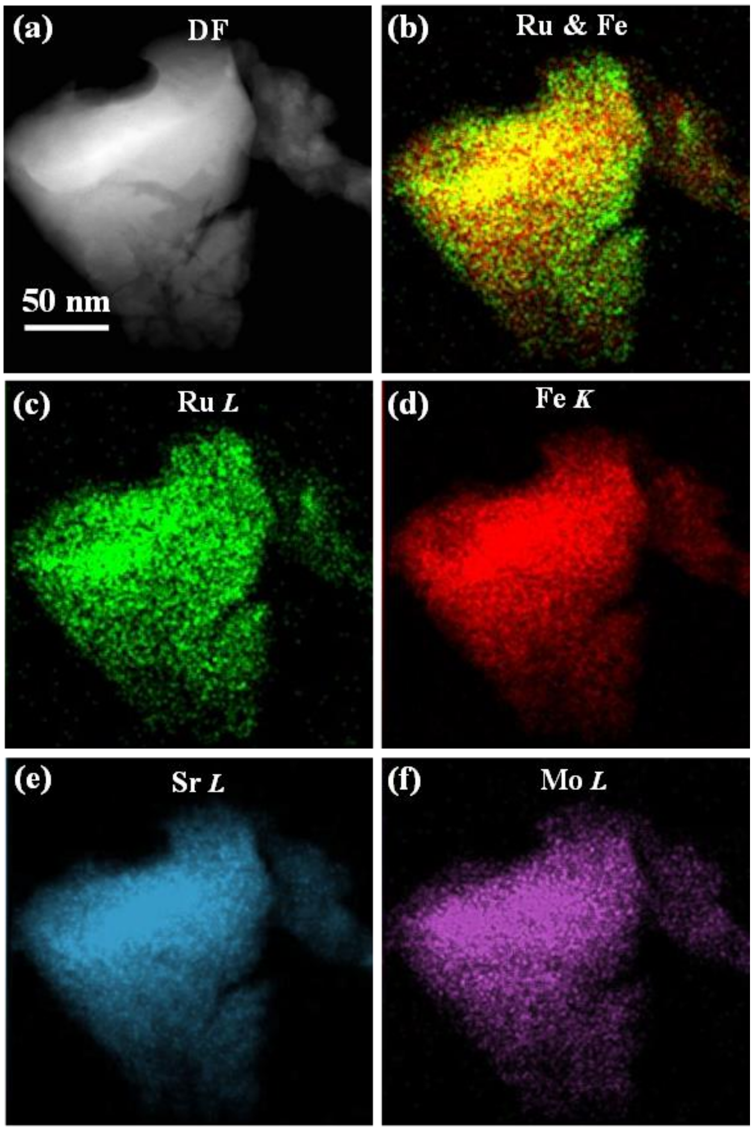

Supplementary Figure 20. STEM-EDS results of SFRuM O3. (**a**) DF-STEM image. (**b-f**) STEM-EDS elemental maps of Sr, Fe, Ru, Mo, and overlapped Ru&Fe.

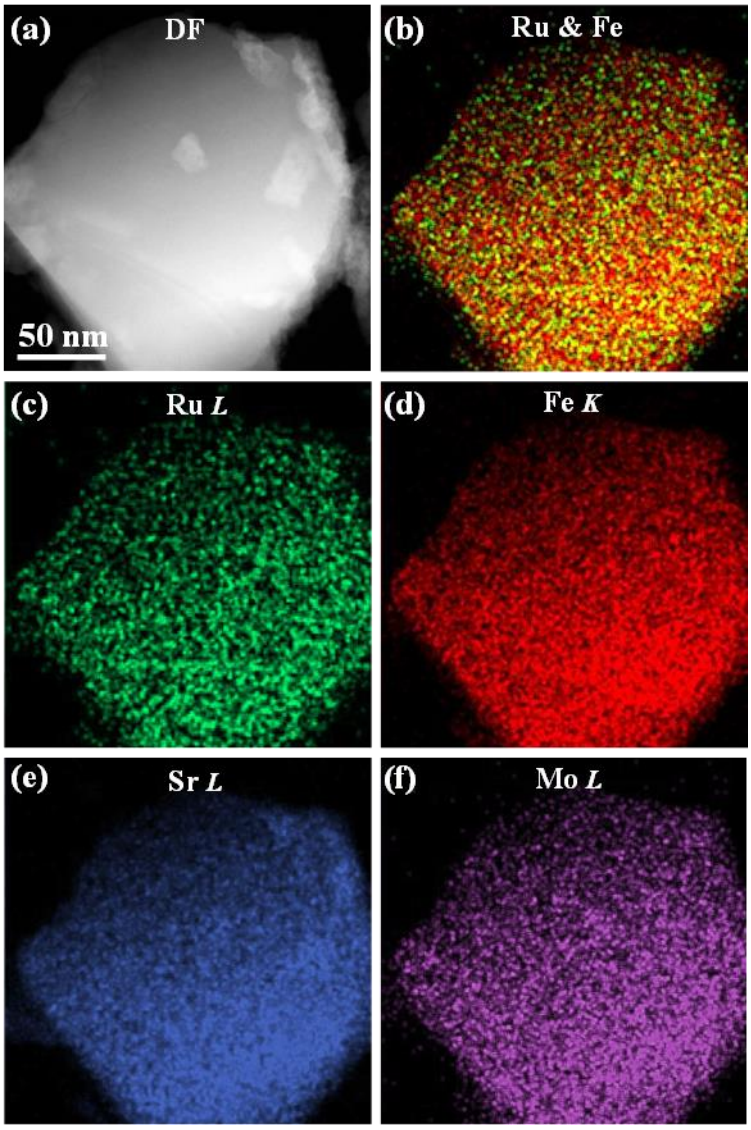

Supplementary Figure 21. STEM-EDS results of the as-prepared SFRuM. (**a**) DF-STEM image. (**b-f**) STEM-EDS elemental maps of Sr, Fe, Ru, Mo, and overlapped Ru&Fe.

Elemental maps of the as-prepared SFRuM clearly showed a homogeneous distribution of Sr, Fe, Ru, and Mo in the perovskite.

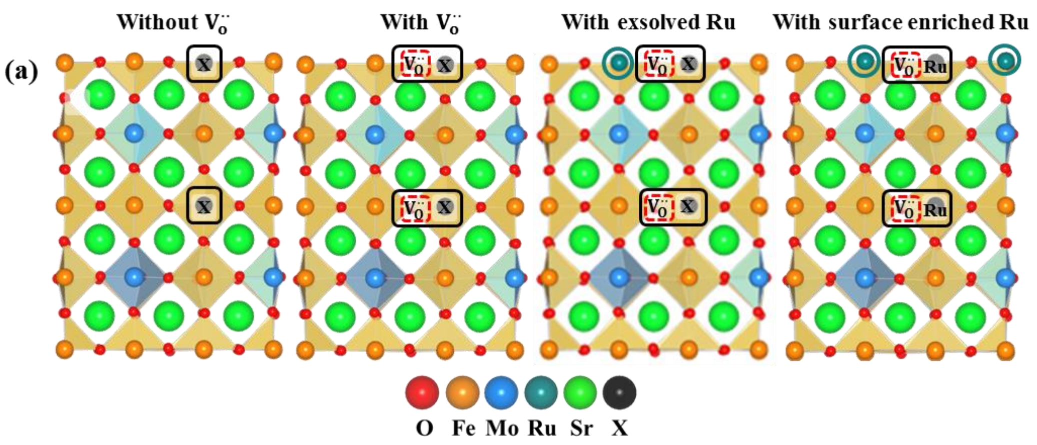

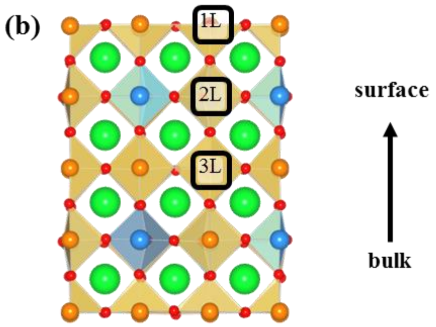

(c)

	T1	T2	T3
1L	**Ru**	Fe	Fe
2L	Fe	**Ru**	Fe
3L	Fe	Fe	**Ru**

Supplementary Figure 22. Segregation effect of individual B-site metal (X). (**a**) Schematic locations of B-site Fe or Ru along SFRuM[010] orientation (side view). From left to right, black frames mark the segregated species without and with oxygen vacancy ($V_O^{\cdot\cdot}$, denoted by red dashed square), with surface exsolved Ru (olive circle) assisted and surface enriched Ru (olive circle) assisted. (**b**) Exchange effect between Fe and Ru, 1L-3L represent the depth away from the outermost surface. For clarity, two spins of Fe atoms are shown only in orange. (**c**) Three types (T1-T3) of Fe/Ru allocations were used to calculate their exchange energies. Black arrow indicates the direction from bulk to surface.

We try to interpret the course of the formation of RuFe alloy: (1) Ru is preferential to segregate onto the surface than preferential ion migration of bulk-biased Ru, which is enhanced by oxygen vacancies and exsolved surface Ru; (2) Then, Fe is reduced onto the surface of Ru NP to produce RuFe intermetallic alloy NPs. This segregation process benefits from the formation of oxygen vacancies, and the surface enriched Ru after redox manipulations as mentioned above.

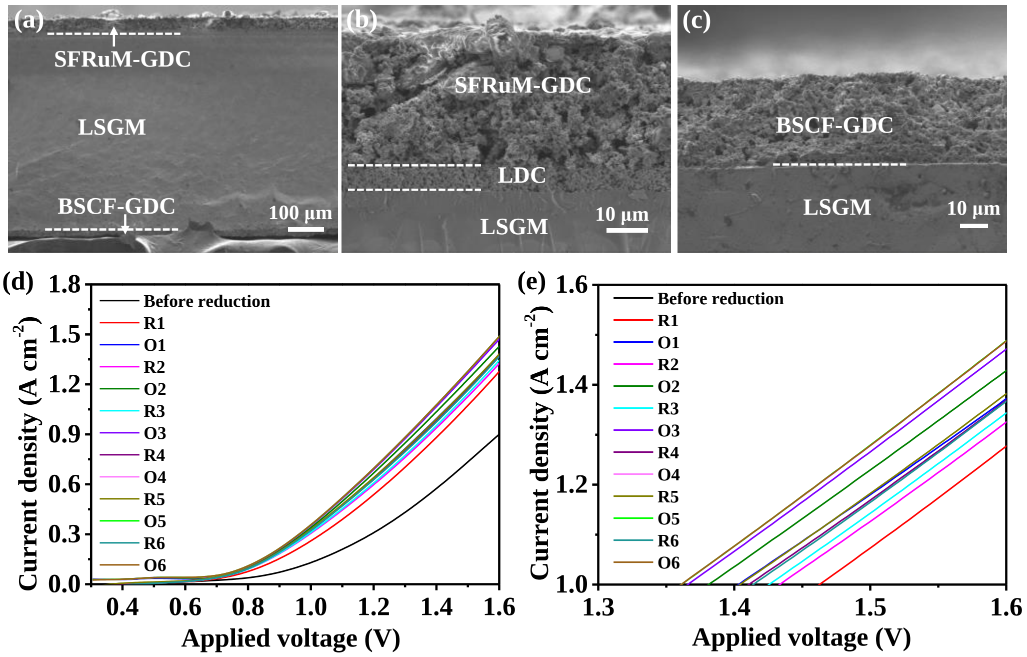

Supplementary Figure 23. Cross-sectional SEM images of (**a**) SFRuM-GDC|LDC|LSGM|BSCF-GDC cell. (**b**) LSGM|LDC supported SFRuM-GDC cathode and (**c**) LSGM supported BSCF-GDC anode. Electrochemical results of CO_2 electrolysis. (**d**) I-V curves of SFRuM-GDC cells before reduction and after redox manipulations at 800 ℃. (**e**) Magnified image of (**a**).

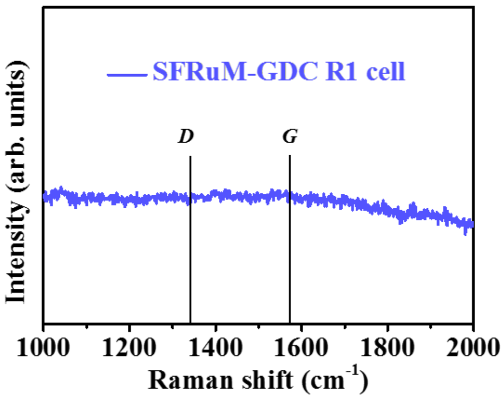

Supplementary Figure 24. Raman spectrum of SFRuM-GDC R1 cell after stability test for 567 h.

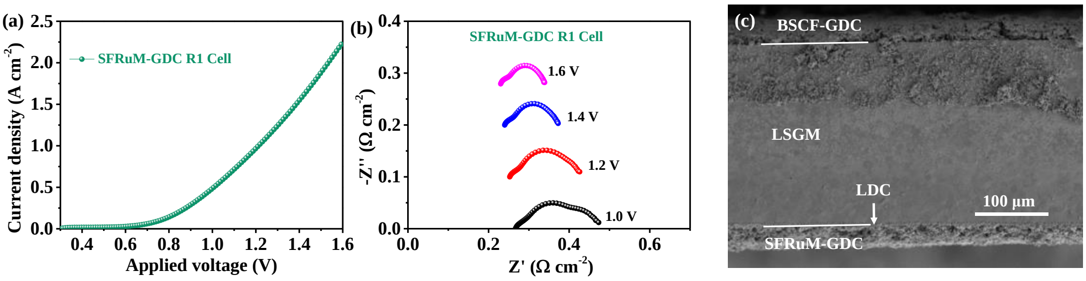

Supplementary Figure 25. (**a**) I-V curves of SFRuM-GDC R1 cells using a thin LSGM electrolyte with a thickness of ~260 μm. (**b**) The corresponding EIS curves at different potentiostatic voltages at 800 ℃. (**c**) Cross-sectional SEM image of SFRuM-GDC|LDC|LSGM|BSCF-GDC cell.

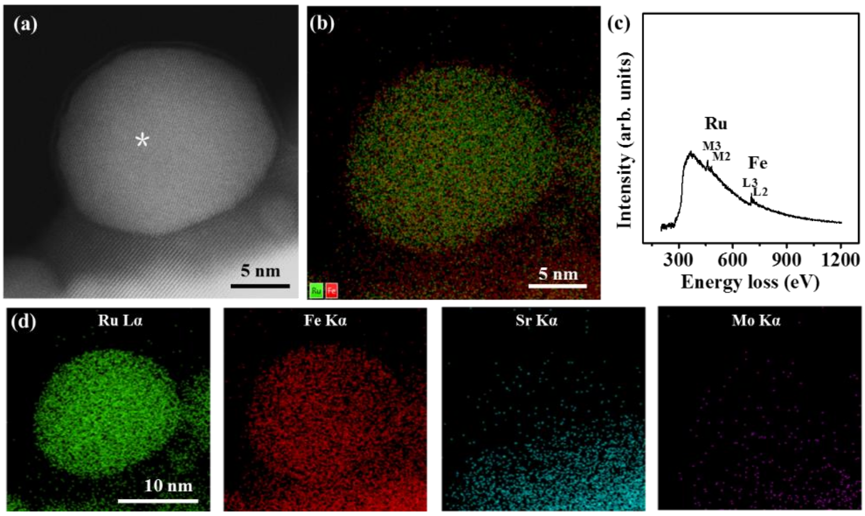

Supplementary Figure 26. In situ STEM results. (**a**) In situ HAADF-STEM image of SFRuM after reduction at 800 ℃ for ~1 h. (**b**,**d**) In situ STEM-EDS elemental maps of (**a**). (**c**) In situ STEM-EELS spectrum in (**a**).

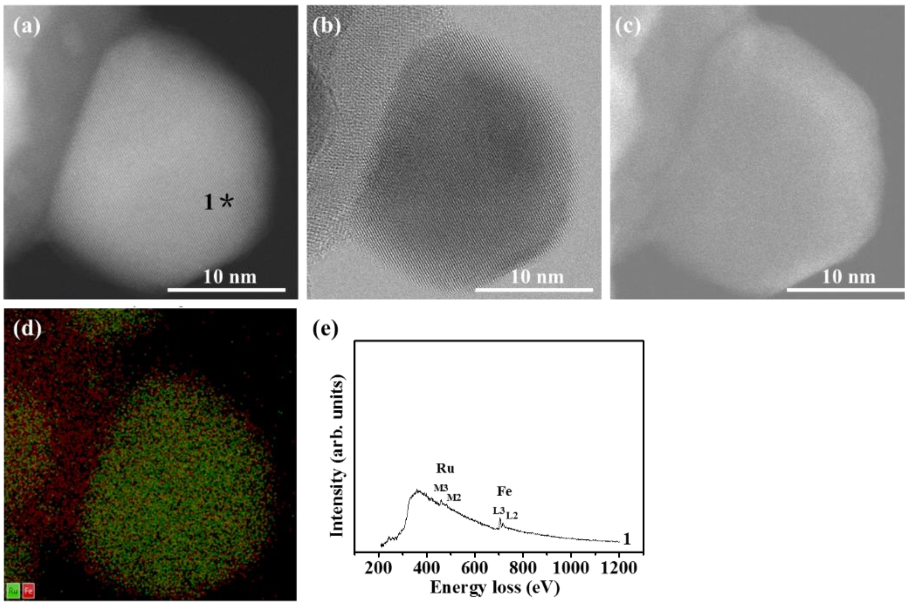

Supplementary Figure 27. (**a-c**) In situ DF, BF and SE-STEM images of SFRuM after in situ reduction at 800 ℃ for ~1 h. (**d**) In situ STEM-EDS elemental map of (**a**).

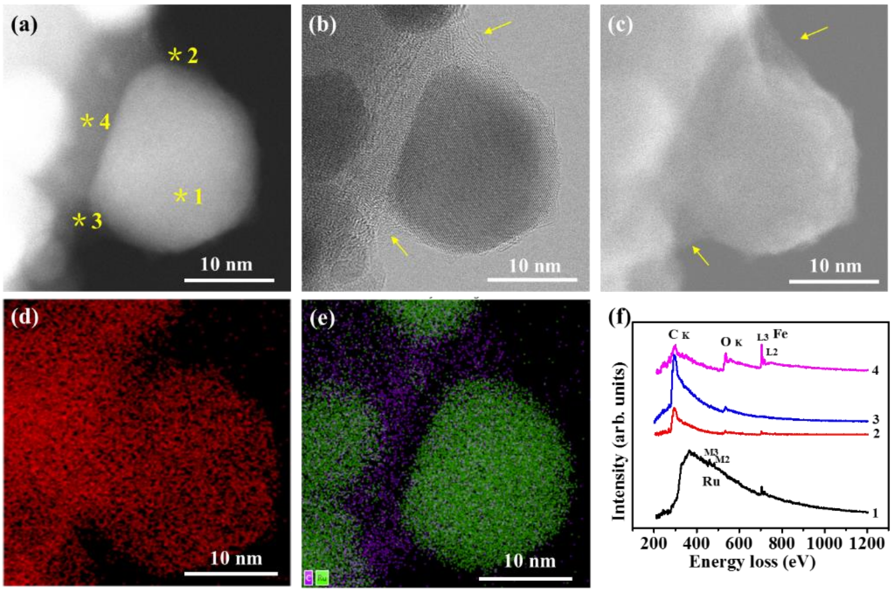

Supplementary Figure 28. (**a-c**) In situ DF, BF and SE-STEM images after exposure to 10 Pa of CO_2 for ~5 min at 200 ℃ of Supplementary Fig. 27. The adsorbed species are indicated by yellow arrows. (**d-e**) In situ STEM-EDS elemental maps of (**a**). (**f**) In situ STEM-EELS spectra of (**a**).

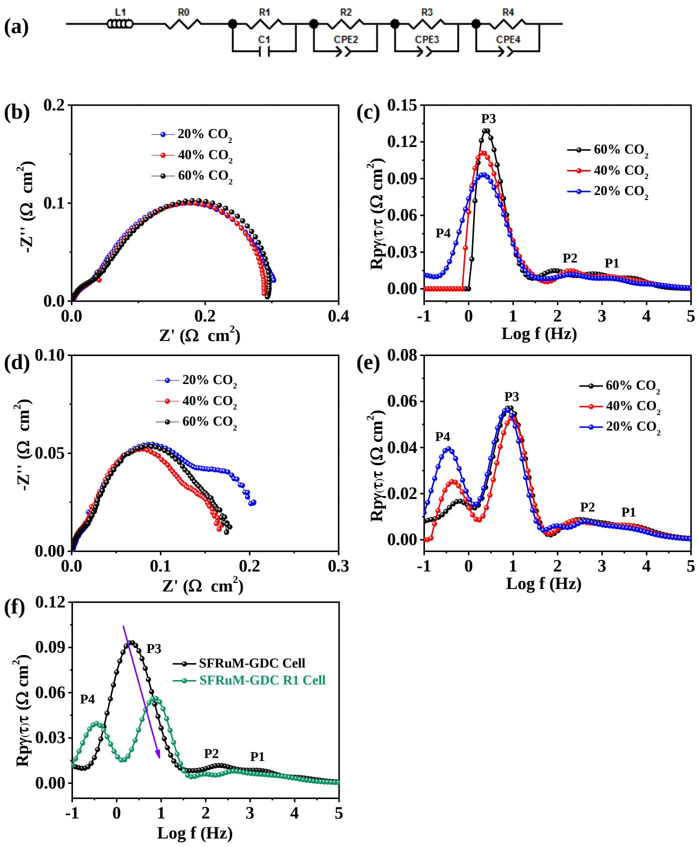

Supplementary Figure 29. (**a**) The established equivalent circuit model for the fitting of the electrochemical impedance spectroscopy (EIS). (**b**) EIS of SFRuM-GDC cell under different partial pressure of CO_2. (**c**) Distribution function of relaxation times (DRT) spectra calculated from (**b**). (**d**) EIS of SFRuM-GDC R1 cell under different partial pressure of CO_2. (**e**) DRT spectra calculated from (**d**). (**f**) Comparison of DRT spectra at 1.2 V and 800 ℃.

To identify the elementary electrode processes in EIS, DRT combined with complex nonlinear least square (CNLS) fit was used for EIS analysis to identify P1-P4 processes[4-6], the polarization resistance of the corresponding electrode process is proportional to the peak area. Supplementary Fig. 29a shows the established equivalent circuit model used for the fitting of EIS in Supplementary Fig. 29b,d, and the DRT results upon different partial pressure of CO_2 are shown in Supplementary Fig. 29c,e. It can be observed that P1-P3 processes are independent on the partial pressure of CO_2, whereas P4 process is greatly affected by the partial pressure, which suggests that P4 represents for the gas diffusion process[7]. After reduction, SFRuM-GDC R1 cell showed improved CO_2 electrolysis performance than SFRuM-GDC cell due to the exsolved RuFe alloy NPs. Comparison of DRT spectra for SFRuM-GDC and SFRuM-GDC R1 cells is shown in Supplementary Fig. 29f. P3 process is greatly accelerated for SFRuM-GDC R1 cell, which is assigned to the CO_2 adsorption and activation process. According to the literatures[4,5,8], the high-frequency processes (P1 and P2) represent for the ion migration and oxygen evolution process, respectively.

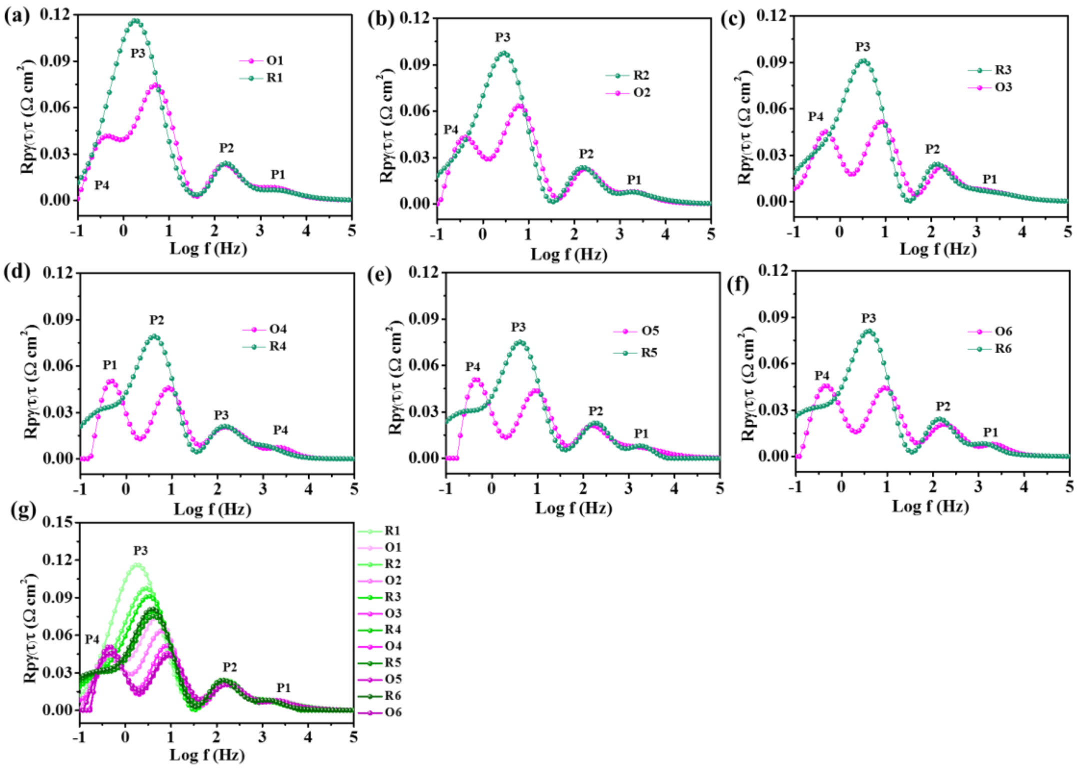

Supplementary Figure 30. The corresponding DRT spectra of SFRuM-GDC cell after redox manipulations in Fig. 5b. (**a**) DRT spectra of SFRuM-GDC R1 and O1. (**b**) DRT spectra of SFRuM-GDC R2 and O2. (**c**) DRT spectra of SFRuM-GDC R3 and O3. (**d**) DRT spectra of SFRuM-GDC R4 and O4. (**e**) DRT spectra of SFRuM-GDC R5 and O5. (**f**) DRT spectra of SFRuM-GDC R6 and O6. (**g**) DRT spectra of SFRuM-GDC R1-O6.

Current density was improved after each reduction and re-oxidation process in the first 3 redox manipulations (Fig. 5a,b). As shown in Supplementary Fig. 30, P1, P2 and P4 processes remain basically unchanged as the same cell with the same electrolyte and anode. Notably, P3 process is accelerated gradually after redox manipulations, suggesting that the improved adsorption and activation capacity is due to the surface enrichment of Ru and gradually enhanced exsolution of RuFe alloy NPs. After 3 redox manipulations (R4-O4-R5-O5-R6-O6), P3 process remains unchanged and the resistance of P3 is reduced after every re-oxidation process (Supplementary Fig. 30e-g), which is due to that the current density can be reproducible on/off on a regular basis (Fig. 5a,b).

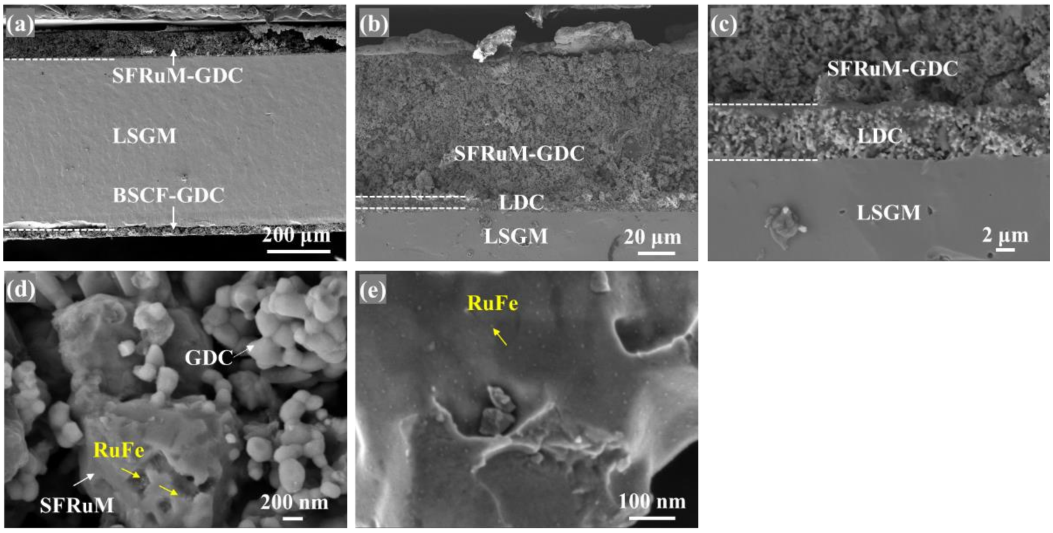

Supplementary Figure 31. Cross-sectional SEM images after 1000 h stability test. (**a**) SFRuM-GDC|LDC|LSGM|BSCF-GDC cell. (**b-c**) LSGM|LDC supported SFRuM-GDC cathode. (**d**) SFRuM-GDC cathode. (**e**) HRSEM image of SFRuM with RuFe alloy NPs.

No delamination was observed in the interface region after 1000 h stability test, moreover, the exsolved RuFe alloy NPs were still uniformly socketed on the SFRuM perovskite without much agglomeration.

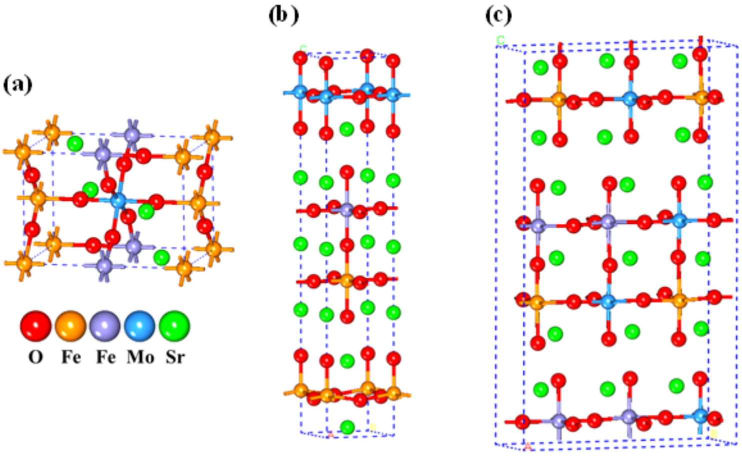

Supplementary Figure 32. Used models for DFT calculations. (**a**) Pristine $Sr_4Fe_3MoO_{12}$ (SFM) cell. (**b**) Pristine $Sr_6Fe_3MoO_{14}$ (RP-SFM) cell. (**c**) Adjusted $Sr_{18}Fe_8Mo_4O_{42}$ supercell with the ratio of Fe : Mo = 2 : 1. Fe atoms with different spins are shown by two colored balls (orange and purple).

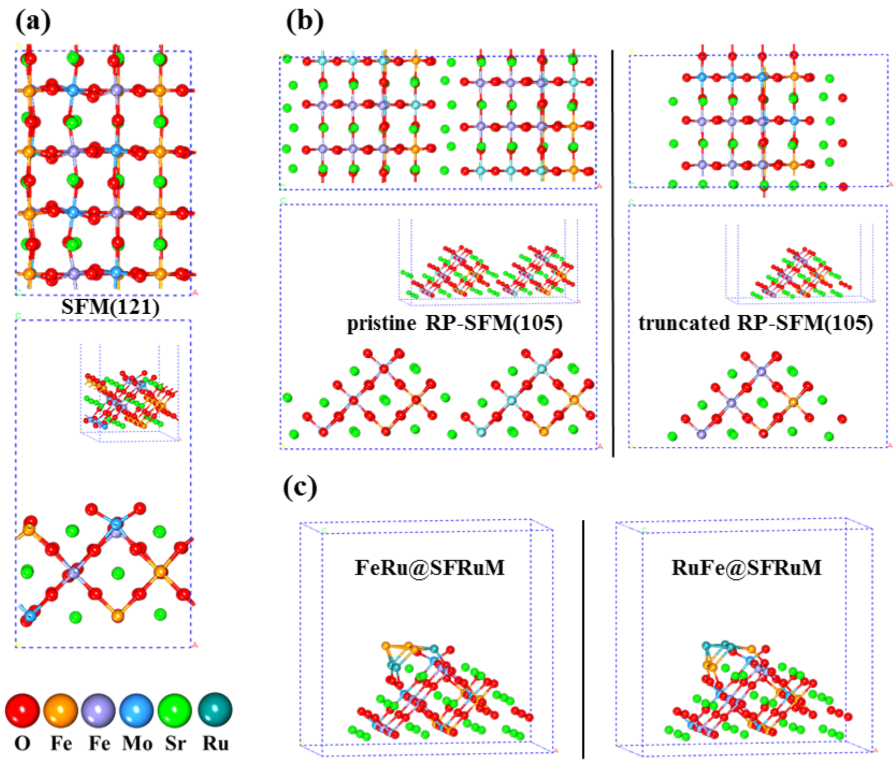

Supplementary Figure 33. Surface and interface models. (**a**) (1 × 2) SFM (121) surface with Fe-O layer terminated, used to model the SFRuM (121) surface. The upper is the top view, and the lower the side view, the inset is the oblique view. (**b**) Pristine (left) and truncated (right) RP-SFM (105) surfaces with Sr-O layer termination. The upper is shown by top view and the bottom is side view, where insets exhibit detailed atomic configurations for clarity. (**c**) Proposed interface structures by placing an Fe_3Ru_3 cluster on the RP-SFM (105) surface. The left is the Ru-terminated interface (FeRu@SFRuM) and the right is Fe-terminated one (RuFe@SFRuM).

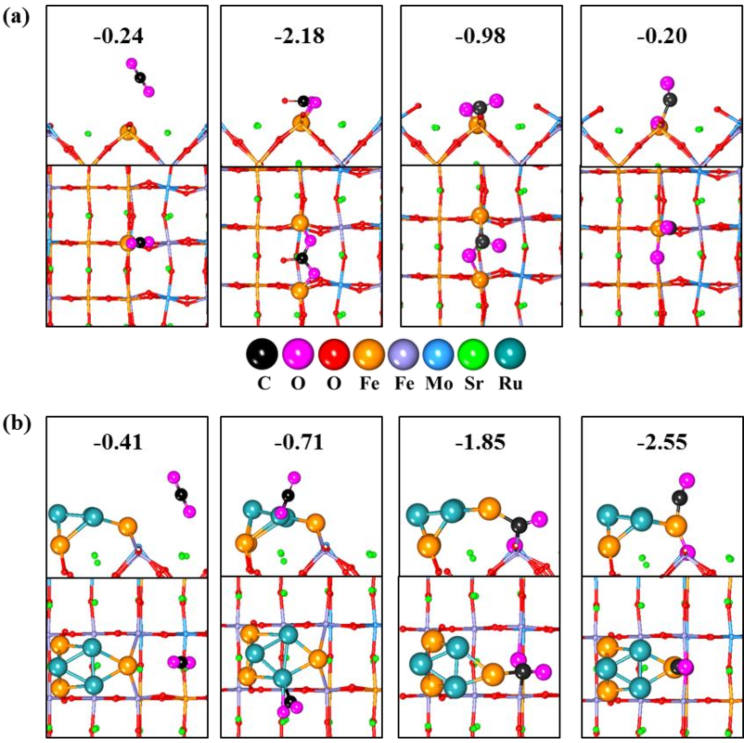

Supplementary Figure 34. Various CO_2 adsorption modes over SFRuM surface and RuFe@SFRuM interface. (**a**) The linear, carbonate-like, bent (bidentate), and dissociated CO_2 adsorption on SFRuM surface (from left to right). (**b**) The linear, two bent (bidentate), and dissociated manners over the RuFe@SFRuM. The adsorption energy here is calculated without thermodynamic corrections.

CO_2 can bind to the active sites in various ways including linear, carbonate-like, bent (bidentate and tridentate), and dissociated adsorptions. Supplementary Fig. 34 displays the linear CO_2* is physisorption (−0.24 eV), the carbonate-like one exhibits a chemisorption (−2.18 eV) with a SFRuM surface reconstruction. These two species are usually the bystanders or poisons for CO_2 electrolysis process. The CO_2* species exhibiting a bidentate or tridentate $CO_2^{\delta-}$ state shall serve as an active intermediate for further decomposition to CO[9], such as the bent adsorption (−0.98 eV) and the dissociated adsorption (−0.20 eV) on the SFRuM surface. For RuFe@SFRuM, the activated $CO_2^{\delta-}$ shows a strong adsorption energy of −1.85 eV at the interface compared with that of −0.71 eV on the NP surface. The result indicates that RuFe@SFRuM interface enhances CO_2 bonding compared to pristine SFRuM surface.

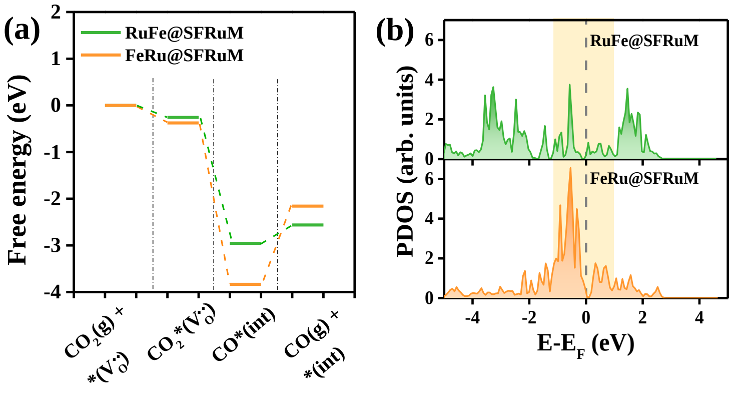

Supplementary Figure 35. CO_2 activation and electronic structure analysis from DFT calculations. (**a**) Gibbs free energy diagram for CO_2 electrolysis process at 800 ℃ and −1.0 V, over two active sites: Fe-terminated (RuFe@SFRuM) and Ru-terminated (FeRu@SFRuM) interfaces. *($V_O^{\cdot\cdot}$) and *(int) denote the oxygen-defected and the intact surface sites. (**b**) Projected density of states (PDOS) of active Fe center of RuFe@SFRuM and Ru center of FeRu@SFRuM corresponding to *(int). The dashed gray line marks the Fermi level and the orange block highlights the difference of the *d*-band.

As shown in Supplementary Fig. 35, Ru-terminated interface merely strengthens the CO_2 adsorption by 0.12 eV than Fe-terminated one. However, CO* desorption, responsible for the rate-determining step (RDS), exhibits a dramatic change from 0.39 eV on RuFe@SFRuM to 1.68 eV of energy releasing on FeRu@SFRuM, which can be also reflected on the electronic structure difference (richer Ru *d*-band occupation at the Fermi level implies more active to adsorption of CO, Supplementary Fig. 35b). This model analysis may provide evidence that the interfacial activity increases with the amount of Fe-terminated architecture. Namely, interfacial Fe coupled with oxygen vacancies not only strengthens CO_2 adsorption but also weakens CO binding, resulting in a high CO_2 electrolysis performance.

Supplementary Table 1. Area intensity ratio by Ru 3*p* and Sr 3*d* of SFRuM before reduction and after redox manipulations.

	SFRuM	R1	O1	R2	O2	R3	O3
Are.(Ru 3*p*)/ Are.(Sr 3*d*)	0.0527	0.0614	0.0684	0.0738	0.0753	0.082	0.120

Supplementary Table 2. Comparison of performance with state-of-the-art electrolyzers using CO_2 as reactant at 800 ℃.

**Cell structure cathode|electrolyte|anode**	**Reference**	**Current density ($A\ cm^{-2}$) (1.6 V)**
LSCM-SDC|YSZ|LSCM-SDC	J. Power Sources 2013, 230, 115	0.11
LSCM-SDC-V_2O_5|YSZ| LSCM-SDC-V_2O_5	Electrochim. Acta 2016, 212, 32	0.25
LSFT|YSZ|LSFT	Electrochem. Commun. 2016, 69, 80	0.32
LSFCr|YSZ|LSFCr	Fuel Cells 2015, 15, 689	0.41
GDC~SFM|YSZ|LSM-YSZ	J. Energy Chem. 2019, 35, 71	0.45
LSTMN|YSZ|LSM	Nat. Commun. 2017, 8:14785	0.54
NiCu-LSCM-SDC |LSGM|LSM-SDC	J. Power Sources 2017, 363, 177	0.78
$FeNi_3$@SFMN-GDC |LSGM|LSCF-GDC	J. Mater. Chem. A 2019, 7, 11967	0.93
Co-Fe-PSFM|YSZ|LSCF-GDC	J. Mater. Chem. A 2016, 4, 17521	1.0 (850 ℃)
CoFe@SFMC-GDC |LSGM|BSCF-GDC	Adv. Mater. 2019, 1906193	1.20
Cu-CMF|ScSZ|LSM-ScSZ	Electrochim. Acta 2017, 235, 365	1.26
F-SFM|LSGM|LSCF-SDC	Adv. Energy Mater. 2018, 1803156	1.36 (1.5 V)
Ni-YSZ|YSZ|SSC-YSZ	RSC Adv. 2016, 6, 112253	1.37
Ni-SDC-YSZ|YSZ| LSM-SDC-YSZ	J. Mater. Chem. A 2015, 3, 15913	1.59
Ni-YSZ|YSZ|Au/YSZ	Angew. Chem. Int. Ed. 2019, 58, 4617	1.65
Fe-Ni-LSFN|YSZ|LSCF-GDC	ACS Catal. 2016, 6, 6219	1.78 (850 ℃)
Ni/$Cr_2O_{3-\delta}$|LSGM|BSCF	J. Power Sources 2019, 430, 20	2.07
NiFe@SFM-SDC |YSZ|LSM-YSZ	J. Mater. Chem. A 2017, 5, 20833	2.16 (1.5 V)
**RuFe@SFRuM-GDC|LSGM| BSCF-GDC**	**This work**	**2.25**
Ni-Fe-LSFM|LSGM|BLC	Adv. Energy Mater. 2015, 5, 1401003	2.32
CoFe@LSCFM-GDC|LSGM| BSCF-GDC	Angew. Chem. Int. Ed. 2020, 59,15968	2.40
Ni-YSZ|YSZ|RuO_2/LBSCF-F	Nano Energy 2019, 57, 186	2.68
Ni/11%MnO_x|LSGM|BSCF	Nat. Commun. 2019, 10, 1550	3.10

LSCM-SDC: $La_{0.75}Sr_{0.25}Cr_{0.5}Mn_{0.5}O_{3-\delta}$-$Sm_{0.2}Ce_{0.8}O_{1.9}$; LSFT: $La_{0.3}Sr_{0.7}Fe_{0.7}Ti_{0.3}O_{3-\delta}$; LSFCr: $La_{0.3}Sr_{0.7}Fe_{0.7}Cr_{0.3}O_{3-\delta}$; SFM: $Sr_2Fe_{1.5}Mo_{0.5}O_{6-\delta}$; LSTMN: $(La_{0.2}Sr_{0.8})_{0.95}Ti_{0.85}Mn_{0.1}Ni_{0.05}O_{3-\delta}$; NiCu-LSCM: $La_{0.75}Sr_{0.25}Cr_{0.5}Mn_{0.5}O_{3-\delta}$; SFMN-GDC: $Sr_2Fe_{0.35}Mo_{0.45}Ni_{0.2}O_{6-\delta}$-$Gd_{0.2}Ce_{0.8}O_{1.9}$; PSFM: $(Pr_{0.4}Sr_{0.6})_3(Fe_{0.85}Mo_{0.15})_2O_7$; SFMC: $Sr_2Fe_{0.35}Mo_{0.45}Co_{0.2}O_{6-\delta}$; Cu-CMF: $Ce_{0.6}Mn_{0.3}Fe_{0.1}O_{2-\delta}$; F-SFM: $Sr_2Fe_{1.5}Mo_{0.5}O_{6-\delta}F_{0.1}$; Fe-Ni-LSFN: $La_{0.6}Sr_{0.4}Fe_{0.8}Ni_{0.2}O_{3-\delta}$; NiFe@SFM: $Sr_{1.9}Fe_{1.5}Mo_{0.4}Ni_{0.1}O_{6-\delta}$; Ni-Fe-LSFM: $La_{0.6}Sr_{0.4}Fe_{0.8}Mn_{0.2}O_3$; LSCFM: $La_{0.4}Sr_{0.6}Co_{0.2}Fe_{0.7}Mo_{0.1}O_{3-\delta}$.

Supplementary Table 3. Comparison of polarization resistances with state-of-the-art electrolyzers using CO_2 as reactant at 800 ℃ and 1.2 V.

**Electrolyzers**	**Reference**	**R_p (Ω cm^{-2})**
LSCM-GDC|YSZ|LSM-YSZ	J. Power Sources 2018, 400, 104	1.5
GDC~SFM|GDC/YSZ|LSM-YSZ	J. Energy Chem. 2019, 35, 71	1.1
LSTMN|YSZ|LSM	Nat. Commun. 2017, 8, 14785	1.0
CMO@LSCM-GDC|YSZ|LSM-YSZ	J. Catal. 2018, 359, 8	0.98
Ce-LSCrF-GDC|GDC/YSZ/GDC| LSCF-GDC	ACS Appl. Mater. Inter. 2016, 8, 6457	0.85 (1.36 V)
LSCM/GDC|YSZ|LSM-YSZ	J. Power Sources 2020, 451, 227743	0.65
Ni-YSZ/YSZ/RuO_2@LSM-YSZ	Energy Storage Mater. 2018, 13, 207	0.60
$FeNi_3$@SFMN-GDC|LDC/LSGM| LSCF-GDC	J. Mater. Chem. A 2019, 7, 11967	0.42
R-LCaFN|YSZ|LCaFN	J. Mater. Chem. A 2020, 8, 14895	0.31 (1.3 V)
LSFT|LSGM|LSCF-GDC	Electrochim. Acta 2020, 342, 136026	0.30
R-LSFN-GDC|GDC/YSZ/GDC| LSFN-GDC	J. CO_2 Util. 2019, 31, 43	0.29
R-LCaFN-GDC|GDC/YSZ/GDC| LCaFN-GDC	J. Power Sources 2020, 475, 22860	0.27
LTFO|LDC/LSGM|LSCF-GDC	J. Mater. Chem. A 2020, 8, 21053	0.20
LBFO|LDC/LSGM|LSCF-GDC LDC/LSGM|LSCF-GDC	J. Power Sources 2019, 485, 229343	0.19
Ni/11%MnO_x|YSZ|LSM-SDC	Nat. Commun. 2019, 10, 1173	0.17
Ni-YSZ/YSZ/LCN-GDC	Nano Energy 2020, 77, 105207	0.16
CoFe@LSCFM-GDC|LDC/LSGM| BSCF-GDC	Angew. Chem. Int. Ed. 2020, 59, 15968	0.15
CoFe@SFMC-GDC|LDC/LSGM| BSCF-GDC	Adv. Mater. 2020, 32, 1906193	0.12
**SFRuM-GDC**	**This work**	**0.32**
**SFRuM-GDC after first reduction**	**This work**	**0.21**
**SFRuM-GDC after 6 redox manipulations**	**This work**	**0.11**

LSCM-GDC: $(La_{0.75}Sr_{0.25})_{0.95}(Cr_{0.5}Mn_{0.5})O_{3-\delta}$-$Ce_{0.8}Gd_{0.2}O_{1.9}$; SFM: $Sr_2Fe_{1.5}Mo_{0.5}O_{6-\delta}$; LSTMN: $(La_{0.2}Sr_{0.8})_{0.95}Ti_{0.85}Mn_{0.1}Ni_{0.05}O_{3-\delta}$; Ce-LSCrF: $La_{0.65}Sr_{0.3}Ce_{0.05}Cr_{0.5}Fe_{0.5}O_{3-\delta}$; LCaFN: $La_{0.6}Ca_{0.4}Fe_{0.8}Ni_{0.2}O_{3-\delta}$; SFMN: $Sr_2Fe_{0.35}Mo_{0.45}Ni_{0.2}O_{6-\delta}$; R-LSFN: $La_{0.6}Sr_{0.4}Fe_{0.8}Ni_{0.2}O_{3-\delta}$; LSFT: $La_{0.3}Sr_{0.7}Fe_{0.9}Ti_{0.1}O_{3-\delta}$; R-LSFN: $La_{0.6}Sr_{0.4}Fe_{0.8}Ni_{0.2}O_{3-\delta}$; LTFO: $La_{0.66}Ti_{0.8}Fe_{0.2}O_{3-\delta}$; LBFO: $La_{0.8}Ba_{0.2}FeO_{3-\delta}$; LCN: $LaCo_{0.6}Ni_{0.4}O_{3-\delta}$; LCN: $LaCo_{0.6}Ni_{0.4}O_{3-\delta}$; LSCFM: $La_{0.4}Se_{0.6}Co_{0.2}Fe_{0.7}Mo_{0.1}O_{3-\delta}$; SFMC: $Sr_2Fe_{0.35}Mo_{0.45}Co_{0.2}O_{6-\delta}$.

Supplementary Table 4. Zero-point energy (*ZPE*), enthalpy (δH), entropy ($-T\Delta S$), and total Gibbs free energy corrections (G-E_{elec}). (unit: eV; T = 800 ℃)

Species	*ZPE*	δH	$-T\Delta S$	G-E_{elec}
CO	0.13	0.34	−2.64	−2.16
CO_2	0.30	0.49	−3.05	−2.26
CO*(Fe-SFRuM)	0.18	0.43	−1.22	−0.61
CO_2*(Fe-SFRuM)	0.29	0.60	−1.45	-0.56
CO*(Ru-SFRuM)	0.22	0.39	−0.97	−0.36
CO_2*(Ru-SFRuM)	0.31	0.58	−1.25	−0.49
CO*(RuFe@SFRuM)	0.20	0.41	−1.09	−0.48
CO_2*(RuFe@SFRuM)	0.25	0.62	−1.54	−0.67
CO*(FeRu@SFRuM)	0.22	0.39	−1.02	−0.41
CO_2*(FeRu@SFRuM)	0.28	0.60	−1.40	−0.52

Supplementary references

1 Zhu, H. *et al.* Constructing Hierarchical Interfaces: TiO_2-Supported PtFe-FeO(x) Nanowires for Room Temperature CO Oxidation. *J. Am. Chem. Soc.* **137**, 10156−10159 (2015).

2 Chen, G. *et al.* Interfacial effects in Iron-Nickel Hydroxide-Platinum nanoparticles enhance catalytic oxidation. *Science* **344**, 495−499 (2014).

3 Li, Y. *et al.* Oxidation-induced segregation of FeO on the Pd-Fe alloy surface. *Appl. Surf. Sci.* **525**, 146484 (2020).

4 Zhang, X. *et al.* Enhancing electrocatalytic CO_2 reduction in solid oxide electrolysis cell with $Ce_{0.9}Mn_{0.1}O_{2-\delta}$ nanoparticles-modified LSCM-GDC cathode. *J. Catal.* **359**, 8−16 (2018).

5 Zhang, X. *et al.* $(La_{0.75}Sr_{0.25})_{0.95}(Cr_{0.5}Mn_{0.5})O_{3-\delta}$-$Ce_{0.8}Gd_{0.2}O_{1.9}$ scaffolded composite cathode for high temperature CO_2 electroreduction in solid oxide electrolysis cell. *J. Power Sources* **400**, 104−113 (2018).

6 Zhang, X. *et al.* Enhanced oxygen reduction activity and solid oxide fuel cell performance with a nanoparticles-loaded cathode. *Nano Lett.* **15**, 1703−1709 (2015).

7 Bian, L. *et al.* Electrochemical performance and stability of $La_{0\ 5}Sr_{0\ 5}Fe_{0\ 9}Nb_{0\ 1}O_{3-\delta}$ symmetric electrode for solid oxide fuel cells. *J. Power Sources* **399**, 398−405 (2018).

8 Lv, H. *et al.* Atomic-scale insight into exsolution of CoFe alloy nanoparticles in $La_{0.4}Sr_{0.6}Co_{0.2}Fe_{0.7}Mo_{0.1}O_{3-\delta}$ with efficient CO_2 electrolysis. *Angew. Chem. Int. Ed.* **59**, 15968−15973 (2020).

9 Zhou, Y. *et al.* Pd single site-anchored perovskite cathode for CO_2 electrolysis in solid oxide electrolysis cells. *Nano Energy* **71**, 104598 (2020).
