# Supplementary material for: Promoting exsolution of RuFe alloy nanoparticles on Sr2Fe1.4Ru0.1Mo0.5O6−δ via repeated redox manipulations for CO2 electrolysis
Source: Nat Commun. 2021 Sep 27;12:5665. doi: 10.1038/s41467-021-26001-8 (PMC8476569; doi:10.1038/s41467-021-26001-8)
Supplement: Supplementary file 3 — Description of Additional Supplementary Files [file 41467_2021_26001_MOESM3_ESM.pdf]

**Description of Additional Supplementary Files**

File Name: Supplementary Movie 1
Description: In situ STEM video of a RuFe@SFRuM interface annealed in 10 Pa $O_2$ at 200 °C.

File Name: Supplementary Movie 2
Description: In situ STEM video of a RuFe@SFRuM interface annealed in 10 Pa $CO_2$ at 200 °C.
